# Spatial Regulation of T-Cell Signaling by Programmed Death-Ligand 1 on Wireframe DNA Origami Flat Sheets

**DOI:** 10.1021/acsnano.0c10632

**Published:** 2021-02-08

**Authors:** Trixy Fang, Jonatan Alvelid, Joel Spratt, Elena Ambrosetti, Ilaria Testa, Ana I. Teixeira

**Affiliations:** † Department of Medical Biochemistry and Biophysics, Karolinska Institute, 171 65 Stockholm, Sweden; ‡ Department of Applied Physics and Science for Life Laboratory, KTH Royal Institute of Technology, 100 44 Stockholm, Sweden

**Keywords:** DNA nanotechnology, DNA origami, PD-1 receptor, cancer immunotherapy, nanoscale spatial distribution

## Abstract

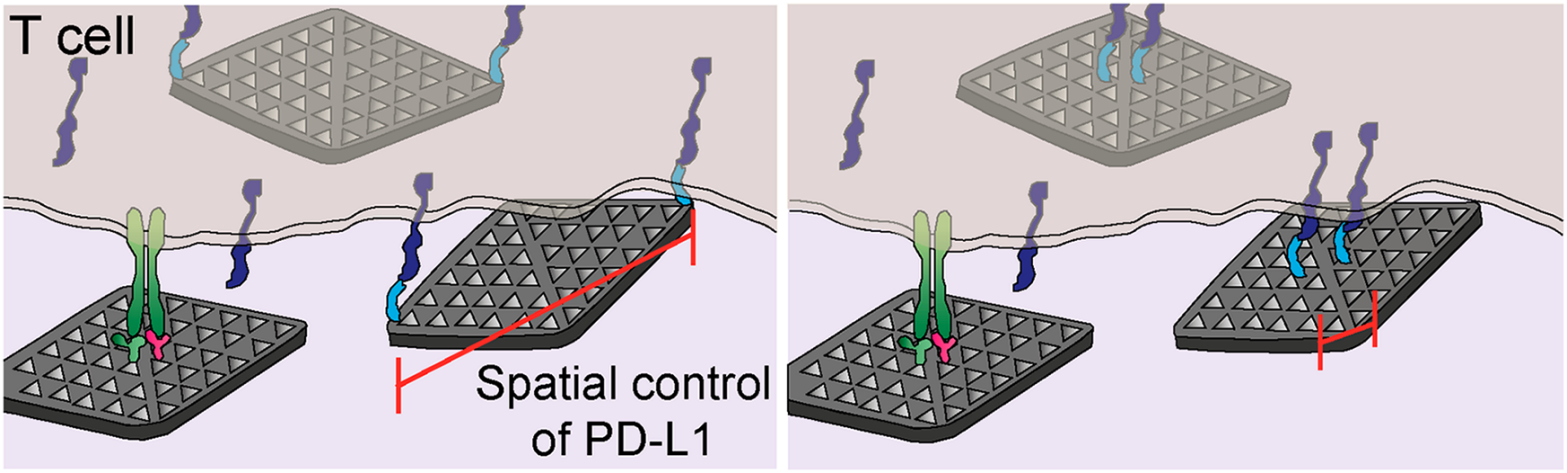

Programmed Death-1 (PD-1) is a coinhibitory receptor expressed on activated T cells that suppresses T-cell signaling and effector functions. It has been previously shown that binding to its ligand PD-L1 induces a spatial reorganization of PD-1 receptors into microclusters on the cell membrane. However, the roles of the spatial organization of PD-L1 on PD-1 clustering and T-cell signaling have not been elucidated. Here, we used DNA origami flat sheets to display PD-L1 ligands at defined nanoscale distances and investigated their ability to inhibit T-cell activation *in vitro*. We found that DNA origami flat sheets modified with CD3 and CD28 activating antibodies (FS-α-CD3-CD28) induced robust T-cell activation. Co-treatment with flat sheets presenting PD-L1 ligands separated by ∼200 nm (FS-PD-L1-200), but not 13 nm (FS-PD-L1-13) or 40 nm (FS-PD-L1-40), caused an inhibition of T-cell signaling, which increased with increasing molar ratio of FS-PD-L1-200 to FS-α-CD3-CD28. Furthermore, FS-PD-L1-200 induced the formation of smaller PD-1 nanoclusters and caused a larger reduction in IL-2 expression compared to FS-PD-L1-13. Together, these findings suggest that the spatial organization of PD-L1 determines its ability to regulate T-cell signaling and may guide the development of future nanomedicine-based immunomodulatory therapies.

Programmed death-1 (PD-1) receptor is one of the most promising immune checkpoint targets for reactivation of the adaptive immune response in cancer immunotherapy.^1,2^ PD-1 acts as a coinhibitory receptor expressed in antigen-stimulated T cells to suppress T-cell signaling.^3−6^ Binding of PD-1 to its ligands PD-L1 or PD-L2 overexpressed in cancer cells and antigen-presenting cells (APC) within the tumor microenvironment represses the antitumor activity of effector T cells.^7−9^ Although blockade of PD-1 and PD-L1/PD-L2 interactions has produced clinical successes, the majority of patients do not benefit from this treatment.^10,11^ Furthermore, PD-1 signaling is also critical for maintaining T-cell tolerance and preventing autoimmunity,^12−14^ and autoimmune adverse events can arise following PD-1 blockade in cancer immunotherapy.^15,16^ Conversely, PD-1 agonists have been proposed as therapies for autoimmune diseases.^17^ Therefore, there is a need to gain a fuller understanding of the mechanisms of PD-1-mediated inhibition of T-cell signaling to improve the effects of PD-1 modulators in the clinic.

Mechanistic studies using supported lipid bilayers to artificially interrogate the T-cell-APC interface (also known as the immunological synapse) have revealed the formation of PD-1 microclusters on the T-cell membrane upon binding to PD-L1.^18−21^ The PD-1 microclusters partially colocalized with T-cell receptor (TCR) microclusters during initial cell-bilayer contact and subsequently migrated to costimulatory receptor CD28 microclusters, resulting in dephosphorylation of TCR-CD3 and CD28 complexes by recruitment of Src homology region 2 domain-containing phosphatase-2 (SHP-2).^18,19^ While some studies have reported that PD-1 is primarily associated with TCR signaling,^6,18,22,23^ others have either shown that CD28 is the preferred target^19,24^ or that both TCR and CD28 are equally inhibited.^25^ Furthermore, the costimulatory receptor CD2 was shown to localize to the outer edge of the immunological synapse and boost TCR signaling, an effect which was dampened by colocalization with ligand-bound PD-1, even in the absence of CD28 engagement.^20^ Although the underlying mechanisms are not fully elucidated, these studies support the hypothesis that the spatial organization of PD-1 on the T-cell membrane is a relevant biophysical regulator for PD-1-mediated T-cell suppression. Interestingly, homodimerization of PD-L1 by nonpeptide-based small molecule inhibitors have been reported to block PD-1/PD-L1 interactions and restore TCR activity.^26−28^ However, a secreted PD-L1 alternative splice variant that forms homodimers was more effective in T-cell inhibition than soluble PD-L1 monomers.^29^ Therefore, the effects of spatial proximity of PD-L1 ligands on PD-1 mediated inhibition of T-cell signaling remain unclear.

DNA origami nanofabrication presents a robust and programmable approach to position individual proteins with nanoscale precision.^30,31^ DNA origami-based nanostructures have been used to produce nanoscale protein patterns that tune membrane receptor activation, downstream signaling and overall cellular responses.^32−35^ TCR activation on bioengineered surfaces commonly utilize antibodies to CD3, a signal transduction component of the TCR complex, for a nonantigen specific trigger.^36−39^ Costimulation with CD28 antibodies provides a secondary signal for T-cell activation which results in cell proliferation and the production of cytokines such as the key pleiotropic lymphocyte mitogen interleukin-2 (IL-2).^39−41^ Here, we used the wireframe-scaffolded DNA origami strategy to produce DNA origami flat sheets that remain stable under physiological ionic strengths, unlike DNA nanostructures composed of densely packed DNA helices.^42,43^ These DNA origami flat sheets were functionalized to present either agonistic anti-CD3 antibodies (FS-α-CD3), antibodies against CD3 and CD28 (FS-α-CD3-CD28), or PD-L1 proteins separated by varying nanoscale distances. As a readout of T-cell activation, we analyzed the activity of a luciferase reporter driven by nuclear factor of activated T cells (NFAT) response elements in PD-1-expressing Jurkat T cells. NFAT is a family of transcription factors that are induced following TCR ligation to elicit transcription of genes involved in T-cell activation and development, including IL-2.^44^ We demonstrated that DNA flat sheets presenting two PD-L1 ligands separated by 200 nm (FS-PD-L1-200), but not 13 nm (FS-PD-L1-13) or 40 nm (FS-PD-L1-40), were able to reduce the levels of CD3- and CD3/CD28-triggered NFAT activity. Moreover, dose–response assays showed that NFAT activity levels decreased with increasing concentrations of FS-PD-L1-200. In addition, cell stimulation with FS-PD-L1-200 resulted in smaller PD-1 cluster sizes and decreased IL-2 mRNA expression compared to stimulation with FS-PD-L1–13. Overall, these findings demonstrate that the nanoscale spatial distribution of PD-L1 tunes T-cell signaling.

## Results and Discussion

### Synthesis of Anti-CD3 IgG-, Anti-CD28 IgG-, and PD-L1–oligo Conjugates

**Figure 1:**
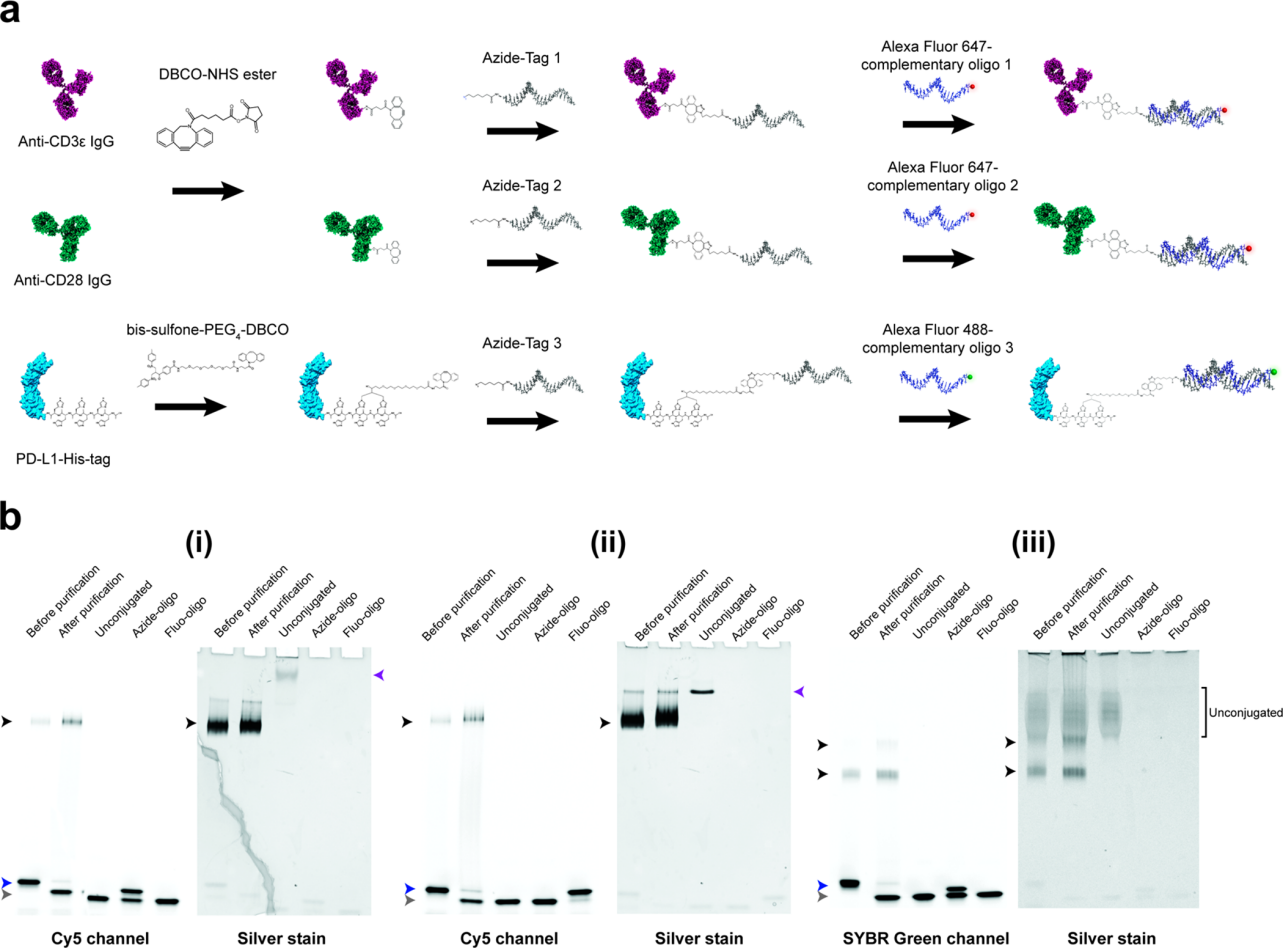
Production of anti-CD3 IgG–, anti-CD28 IgG–, and PD-L1–oligo conjugates. (a) A schematic workflow of anti-CD3ε IgG, anti-CD28 IgG, and PD-L1-His conjugations to 3′ azide oligos *via* copper-free click chemistry and hybridization with complementary fluorescently labeled oligos for fluorescence native PAGE. The antibodies and PD-L1-His were labeled using DBCO-NHS ester and *bis*-sulfone-PEG_4_-DBCO cross-linkers, respectively. (b) Fluorescence native PAGE of (i) anti-CD3 IgG–, (ii) anti-CD28 IgG–, and (iii) PD-L1–oligo conjugates before and after purification, imaged using Cy5 or SYBR Green channels and then silver stained. Unconjugated proteins and azide–oligos mixed with fluorescently labeled oligos were run as controls (purple and blue arrowheads respectively). The control for fluorescently labeled oligos alone (Fluo-oligo) shows unhybridized fluorescently labeled oligos (gray arrowhead). Black arrowheads indicate protein–oligo conjugate products, in which two PD-L1–oligo conjugate products of one and two oligos attached were formed (upper and lower bands, respectively (iii)).

In order to assemble proteins on DNA origami flat sheets, we conjugated anti-CD3 IgG, anti-CD28 IgG, and recombinant PD-L1-His-tag protein to single-stranded DNA oligos (ssDNA) designed to hybridize to complementary oligos that protrude from the origami surface. The antibodies were first functionalized with a dibenzocyclooctyne-*N*-hydroxysuccinimide (DBCO-NHS) ester cross-linker that reacts with primary amine (NH_2_) groups on the antibodies (Figure 1a). Functionalization of PD-L1-His was achieved by a site-specific *bis*-alkylation conjugation at the 6-histidine-tag using a *bis*-sulfone-polyethylene glycol 4-dibenzocyclooctyne (*bis*-sulfone-PEG_4_-DBCO) linker, as previously reported.^33,45^ In a consecutive copper-free click reaction, azide-modified oligos, namely Tag 1, 2, and 3 (Table S1), were then added to react with the DBCO groups on anti-CD3 IgG, anti-CD28 IgG, and PD-L1 respectively. The protein–oligo conjugates were then visualized on either fluorescence native polyacrylamide gel electrophoresis (native PAGE), when hybridized with complementary fluorescently labeled oligos, or by reducing SDS-PAGE (Figure 1b, Figure S1 and S2). Protein–oligo conjugates were confirmed by the presence of higher molecular weight fluorescent bands which were also labeled by silver staining on the native PAGE. The degree of conjugation was found to be between one and two azide–oligos to each protein (Figure 1b and Figure S2), and reduction of antibody–oligo conjugates revealed that these modifications occurred primarily on the heavy chains (Figure S2). The fluorescence native PAGE analysis also showed that the majority of unreacted azide–oligos were removed during the purification step (Figure 1b(i–iii) and Figure S1). Taken together, these results validate the labeling density and quality of the three protein–oligo conjugates used in this study.

### Production of Antibody- and PD-L1-DNA Flat Sheets

As a platform for assembling the protein–oligo conjugates, we used a wireframe DNA origami flat sheet design (Figure S3) which yields nanostructures that have the capacity to fold and remain stable under physiological salt conditions.^43^ The flat sheets were first folded using short ssDNA oligos (staples) which bind complementarily to regions of the p8064 phage ssDNA scaffold. Protein–oligo conjugates were then assembled onto flat sheets *via* hybridization with complementary 5′ ends of staples at designated positions that protrude out of the nanostructure (Figure S3b). Each protein binding site consists of a pair of protruding staples within a 3-tessellation triangulated tile to ensure a high yield of hybridized protein–oligo conjugates at each site.

**Figure 2:**
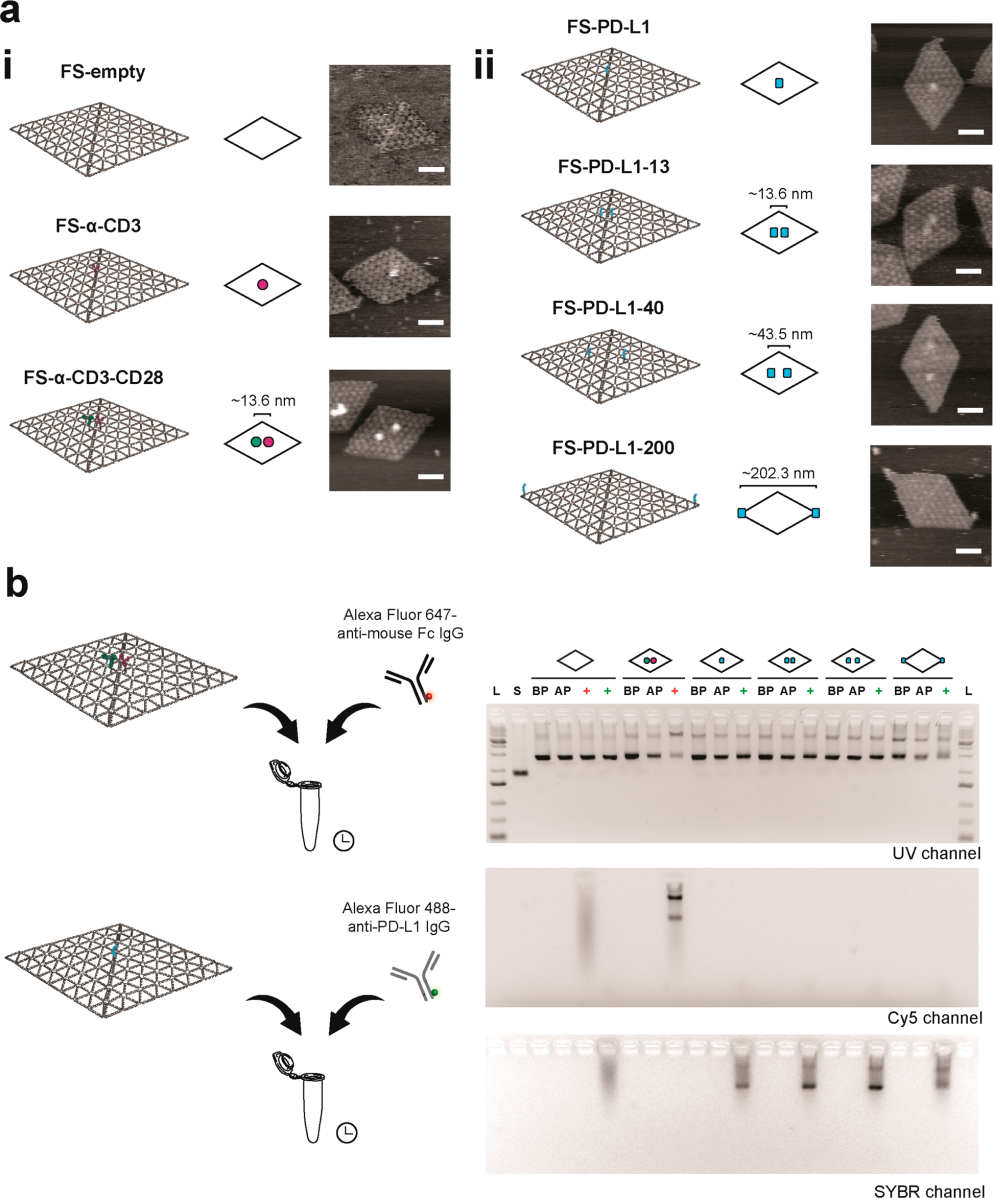
Production of protein–DNA flat sheets. (a) (i) Schematic designs of DNA flat sheets without proteins (FS-empty), functionalized with one anti-CD3 IgG–oligo conjugate in the center (FS-α-CD3), anti-CD3 IgG– and anti-CD28 IgG–oligo conjugates (FS-α-CD3-CD28), and (ii) functionalized with PD-L1–oligo conjugates at different positions (FS-PD-L1, FS-PD-L1-13, FS-PD-L1-40 and FS-PD-L1-200). For simplistic representation, flat sheets are depicted as rhombi and anti-CD3 IgG, anti-CD28 IgG, and PD-L1 are shown as magenta, green, and cyan blobs, respectively. Representative AFM images of flat sheets folded in 1× PBS (right column). Scale bar = 50 nm. (b) Immunolabeling of protein–DNA flat sheets with fluorescently labeled antibodies and agarose gel electrophoresis. L, 1 kb Plus DNA ladder. S, p8064 ssDNA scaffold. BP, before Sepharose purification. AP, after Sepharose purification. Red plus symbol, addition of Alexa Fluor 647-anti-mouse Fc IgG to flat sheets. Green plus symbol, addition of Alexa Fluor 488-anti-PD-L1 IgG to flat sheets.

Using this principle, we developed a panel of DNA flat sheets displaying antibody– and PD-L1–oligo conjugates at different positions (Figure 2). DNA flat sheets without any proteins (FS-empty), with one binding site in the center for anti-CD3 IgG (FS-α-CD3), and with anti-CD3 and anti-CD28 IgGs separated along the helical axis 13.6 nm (FS-α-CD3-CD28), were used as controls (Figure 2a(i)). For flat sheets containing PD-L1, we designed a single PD-L1 binding site in the center (FS-PD-L1) or two binding sites spaced 13.6, 43.5, and 202.3 nm (FS-PD-L1-13, FS-PD-L1-40, and FS-PD-L1-200) (Figure 2a(ii)). The 13.6 nm spacing was designed to display two closely spaced PD-L1 ligands. The 43.5 nm distance was created from adjoining triangle tiles to the 13.6 nm design to control the spatial distribution of PD-L1 ligands within the 35–70 nm range of TCR nanoclusters.^46^ Finally, the 202.3 nm distance was selected to space proteins at the maximum limit that can be achieved with these flat sheets. Atomic force microscopy (AFM) confirmed the self-assembly of flat sheets presenting the protein–oligo conjugates according to design, with estimated fractions of 40–65% (Figures S4–S10). As the protein–oligo conjugates are tethered to the flat sheets *via* a 19 bp or 21 bp oligo, we observed fluctuations in protein distances, which we quantified for FS-α-CD3-CD28 and FS-PD-L1-40 (Figures S11 and S12). For FS-PD-L1-200, the PD-L1 proteins presented at the edge of the origami tended to land on the mica surface and appear as small protrusions on the AFM images (Figures S10 and S13). In addition to AFM imaging, we immunolabeled the protein flat sheets and visualized with agarose gel electrophoresis to verify hybridization of protein–oligo conjugates to the flat sheets (Figure 2b). We observed that flat sheets functionalized with PD-L1 were recognized by Alexa Fluor 488 anti-PD-L1 IgG. Similarly, Alexa Fluor 647-anti-mouse Fc IgG detected flat sheets functionalized with α-CD3 and α-CD28 IgG and increased aggregation of flat sheets (Figure 2b, Cy5 channel). We further characterized the binding ability of flat sheets functionalized with two PD-L1 proteins (FS-PD-L1-13, FS-PD-L1-40, and FS-PD-L1-200) to PD-1 receptors by surface plasmon resonance (SPR) (Figure S14). FS-PD-L1-13, FS-PD-L1-40, and FS-PD-L1-200 exhibited similar binding to PD-1, indicating that the conjugation and hybridization to the flat sheets did not interfere with the binding ability of PD-L1. Together, the AFM imaging, agarose gels and SPR data show that the protein flat sheets were produced according to design and that PD-L1 presented by the flat sheets retained the ability to bind PD-1.

### Spatial Organization of PD-L1 Modulates T-Cell Signaling

**Figure 3:**
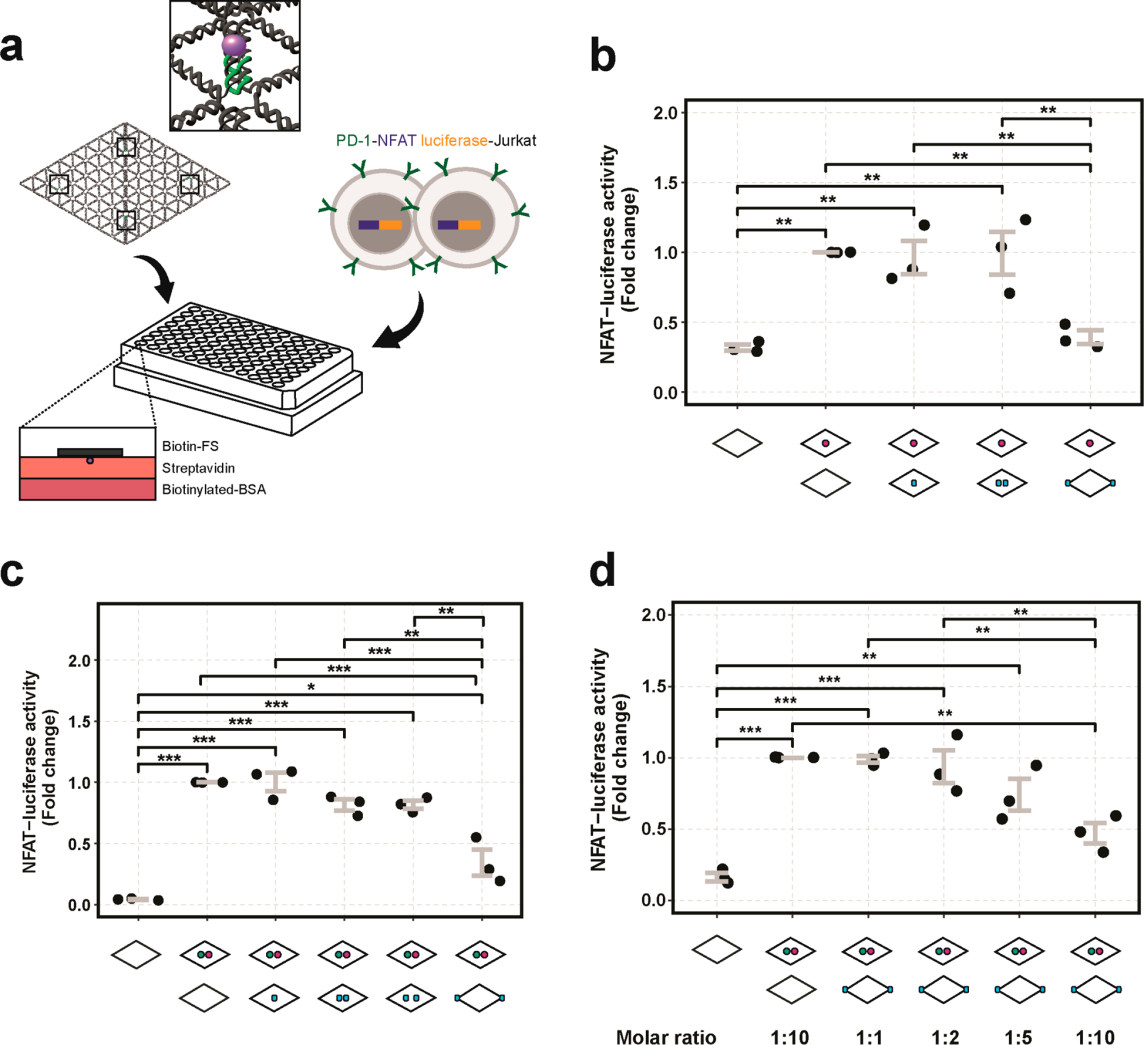
NFAT-luciferase activity regulated by antibody- and PD-L1-flat sheets. (a) Illustration of the experimental setup of protein–DNA flat sheet stimulation of PD-1-NFAT-luciferase reporter Jurkat cells. Flat sheets folded with biotinylated staples, as indicated by the four black squares and a magnified view of a biotin(purple)–oligo(green), were bound to a precoated streptavidin–biotinylated-BSA surface in a 96-well tissue culture plate before cell seeding. (b) Fold changes of NFAT-luciferase activity of cells after stimulation with FS-empty, FS-α-CD3, and costimulation with FS-α-CD3 and FS-PD-L1, FS-PD-L1-13 or FS-PD-L1-200. (c) Fold changes of NFAT-luciferase activity of cells after stimulation with FS-empty, FS-α-CD3-CD28, and costimulation with FS-α-CD3-CD28 and FS-PD-L1, FS-PD-L1-13, FS-PD-L1-40, or FS-PD-L1-200. (d) Fold changes of NFAT-luciferase activity of cells after stimulation with FS-empty, FS-α-CD3-CD28, and increasing molar ratios of FS-α-CD3-CD28 to FS-PD-L1-200 (1:1 to 1:10). Data is presented from three independent experiments (*N* = 3) as indicated by the black dots. Every dot represents the average from triplicates consisting of 3000 cells each. Error bars indicate standard error of the mean. *P* values are indicated as * < 0.05, ** < 0.01, *** < 0.001 as determined by one-way analysis of variance (ANOVA) followed by Tukey’s honest significance (HSD) test.

To investigate the effects of PD-L1 nanoscale spatial distribution on T-cell signaling, we performed a NFAT-luciferase reporter assay in PD-1 expressing Jurkat T cells. To immobilize the flat sheets on the surface, we incorporated biotin-modified staples at four positions in the flat sheets such that the biotins protruded from the non-protein side (Figure 3a). The biotinylated protein flat sheets were then coated on a streptavidin–biotinylated-bovine serum albumin (BSA) surface before cell stimulation. We verified the presence of biotins on the flat sheets with fluorescently labeled streptavidin (Figure S15). Given that NFAT-dependent gene expression can be activated by TCR-CD3 stimulation alone,^47,48^ we first stimulated PD-1-NFAT luciferase cells with flat sheets functionalized only with anti-CD3 antibody (FS-α-CD3) and measured the activation levels with increasing flat sheet concentrations (Figure S16). The NFAT-luciferase activity showed a dose-dependent response with increasing concentrations of FS-α-CD3 stimulation. In subsequent luciferase assays, we used a concentration of 0.5 nM FS-α-CD3 to obtain an activation of NFAT above background levels that could still be regulated by PD-L1 stimulation (Figure 3b). The wells were first coated with FS-α-CD3 and then PD-L1-flat sheets at 10-fold (FS-PD-L1-13, FS-PD-L1-200 at 5 nM) or 20-fold (FS-PD-L1 at 10 nM) molar excess of FS-α-CD3, to obtain equivalent molar amounts of PD-L1. To ensure that the total molar concentration of flat sheets was equivalent between conditions, FS-empty was added in FS-α-CD3, FS-PD-L1-13 and FS-PD-L1-200 conditions.

We found that FS-PD-L1-200 resulted in a considerable decrease in CD3-mediated NFAT-luciferase activity (∼60.8%) which was significantly different from FS-PD-L1 and FS-PD-L1-13 (Figure 3b). FS-PD-L1 and FS-PD-L1-13 showed no significant decrease in CD3-induced NFAT-luciferase activity, indicating that a single PD-L1 ligand or two PD-L1 ligands presented in proximity did not elicit PD-1-inhibition on TCR-CD3 signaling. To further investigate the role of PD-L1 nanoscale spatial distribution on T-cell activation in the presence of costimulation mediated by CD28, we treated cells with flat sheets functionalized with both CD3 and CD28 antibodies (FS-α-CD3-CD28) (Figure 3c). We also introduced FS-PD-L1-40 in the panel of PD-L1-flat sheet designs.

Consistent with the results obtained with FS-α-CD3, FS-PD-L1-200 induced a statistically significant inhibition of NFAT-luciferase activity. In addition, FS-PD-L1-40 showed similar NFAT-luciferase levels to those of FS-PD-L1-13, with no significant decrease compared to cells treated with FS-α-CD3-CD28 alone. Overall, the results confirm that FS-PD-L1-200, but not FS-PD-L1, FS-PD-L1-13, or FS-PD-L1-40, enabled inhibition of CD3- and CD3/CD28-mediated T-cell activation. We also verified the stability of flat sheets in cell culture media by agarose gel electrophoresis. The flat sheets remained largely intact in complete media after 3 h at 37 °C (Figure S17), indicating that the nanostructures were preserved under these cell stimulation conditions.

To investigate the effects of stoichiometry of FS-PD-L1-200 on NFAT-luciferase activity, we stimulated cells with increasing molar ratios of FS-α-CD3-CD28 to FS-PD-L1-200 (1:1 to 1:10). We observed a gradual decrease in NFAT-luciferase activity with increasing molar ratios and a significant reduction at 1:10 ratio of FS-α-CD3-CD28 to FS-PD-L1-200 (Figure 3d). A 1:1 ratio of FS-α-CD3-CD28 to FS-PD-L1-200 showed no changes in NFAT-luciferase activity, indicating that a high stoichiometry of FS-PD-L1-200 is required to elicit PD-1 downregulation of TCR activity in this system. On the other hand, FS-PD-L1 which contains a single PD-L1 protein, showed no significant effect on NFAT-luciferase activity even at high concentrations (Figure S18), suggesting that a high molar ratio of PD-L1 to TCR-activating antibodies is not sufficient for PD-L1 mediated inhibition of T-cell signaling.

### PD-L1-Flat Sheets Modulate PD-1 Nanocluster Size

We next sought to investigate the nanoscale distribution of PD-1 receptors on the cell membrane upon treatment with PD-L1-flat sheets using stimulated emission depletion (STED) microscopy. As a cell model for these studies, we generated a stable Jurkat cell line expressing PD-1 fused with an intracellular self-labeling protein, SNAP-tag, which allows covalent attachment of fluorophore-coupled SNAP ligands. We confirmed PD-1-SNAP expression and PD-1 downregulation of IL-2 secretion in these cells (Figure S19). Consistent with the findings from the NFAT-luciferase assays, FS-PD-L1-200 stimulation of PD-1-SNAP cells resulted in a significant decrease in IL-2 gene expression (Figure S20). These results confirmed that FS-PD-L1-200 tunes TCR downstream signaling by inhibition of IL-2 expression.

**Figure 4:**
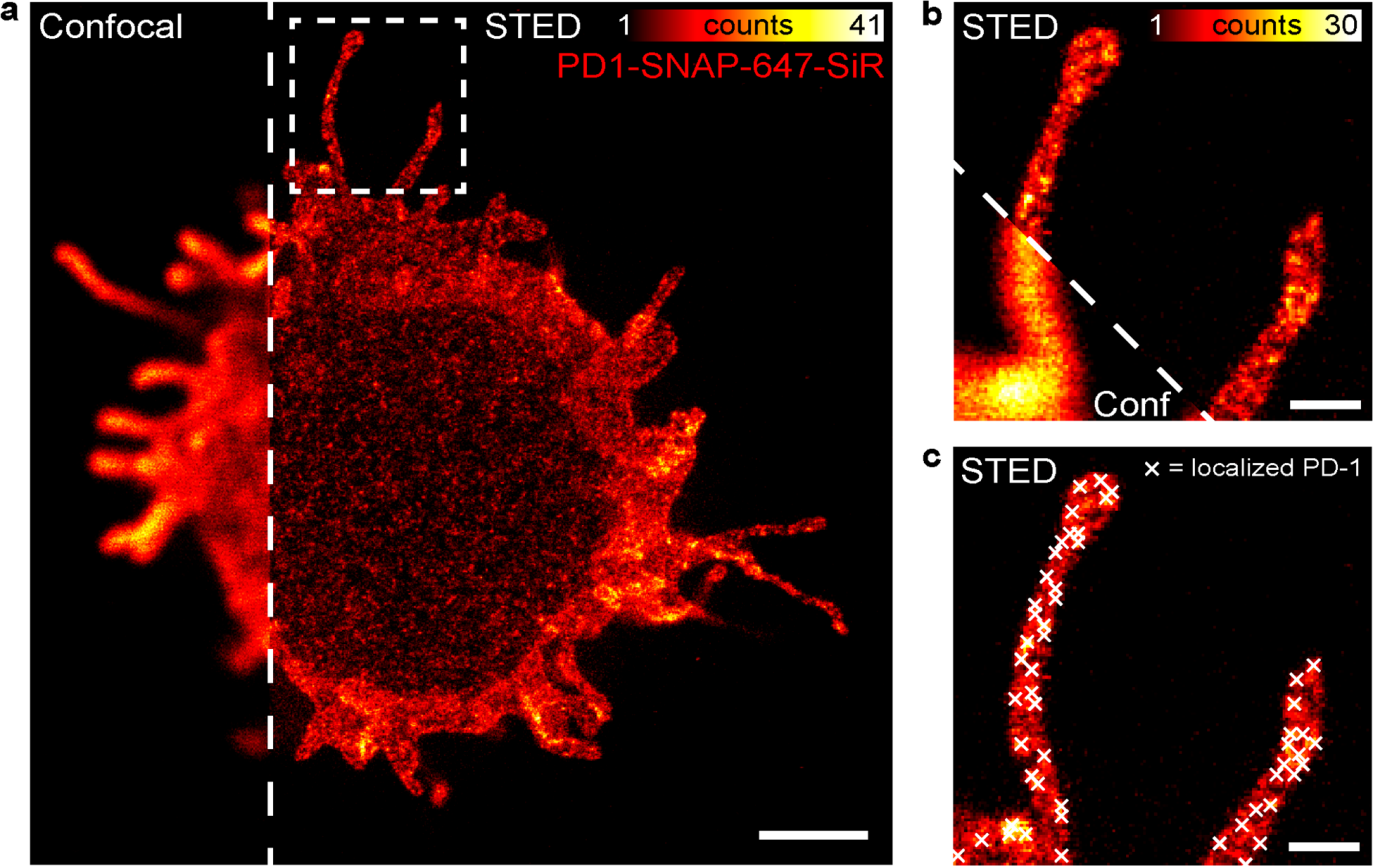
STED microscopy of PD-1 nanoclusters. (a) Confocal *versus* STED image of a PD-1-SNAP Jurkat cell labeled with SNAP-647-SiR. Scale bar = 2 μm. (b) Magnification of the marked area in (a), showing a confocal *versus* STED image of two protrusions with PD-1 localized on the membrane. Scale bar = 500 nm. (c) Same image as in (b) in STED mode with overlaying PD-1 cluster localizations (white crosses). Scale bar = 500 nm.

For the STED imaging experiments, we used the same molar ratios of antibody- and PD-L1-flat sheets as in the NFAT-luciferase assays (0.5 nM FS-α-CD3-CD28, 5 nM FS-PD-L1-13, and 5 nM FS-PD-L1-200) to stimulate PD-1-SNAP cells. To analyze the PD-1 receptor distribution when it is not stimulated by PD-L1, cells were stimulated with 0.5 nM FS-α-CD3-CD28 and 5 nM FS-empty. We first fluorescently labeled the flat sheets to study the distribution of antibody- and PD-L1-flat sheets on the glass surface by confocal microscopy (Figure S21). The negative correlation observed between the antibody- and PD-L1-flat sheets confirmed that they did not overlap each other. We next labeled PD-1-SNAP cells with a cell membrane-permeable SNAP-Cell 647-SiR substrate and stimulated them with antibody- and PD-L1-flat sheets. PD-1-SNAP receptor distribution was imaged by depleting 647-SiR with a 775 nm depletion beam in a far-red custom-built STED system, featuring a spatial resolution within the range of 30–50 nm (Figure 4 and Figure S22). When compared with the corresponding confocal images, PD-1 receptor clusters were only localizable using STED microscopy (Figure 4a,b). To localize these PD-1 receptor clusters (Figure 4c), we followed the data preprocessing and localization pipeline as described in Figure S23. Using the localized cluster coordinates, we performed three kinds of analyses.

**Figure 5:**
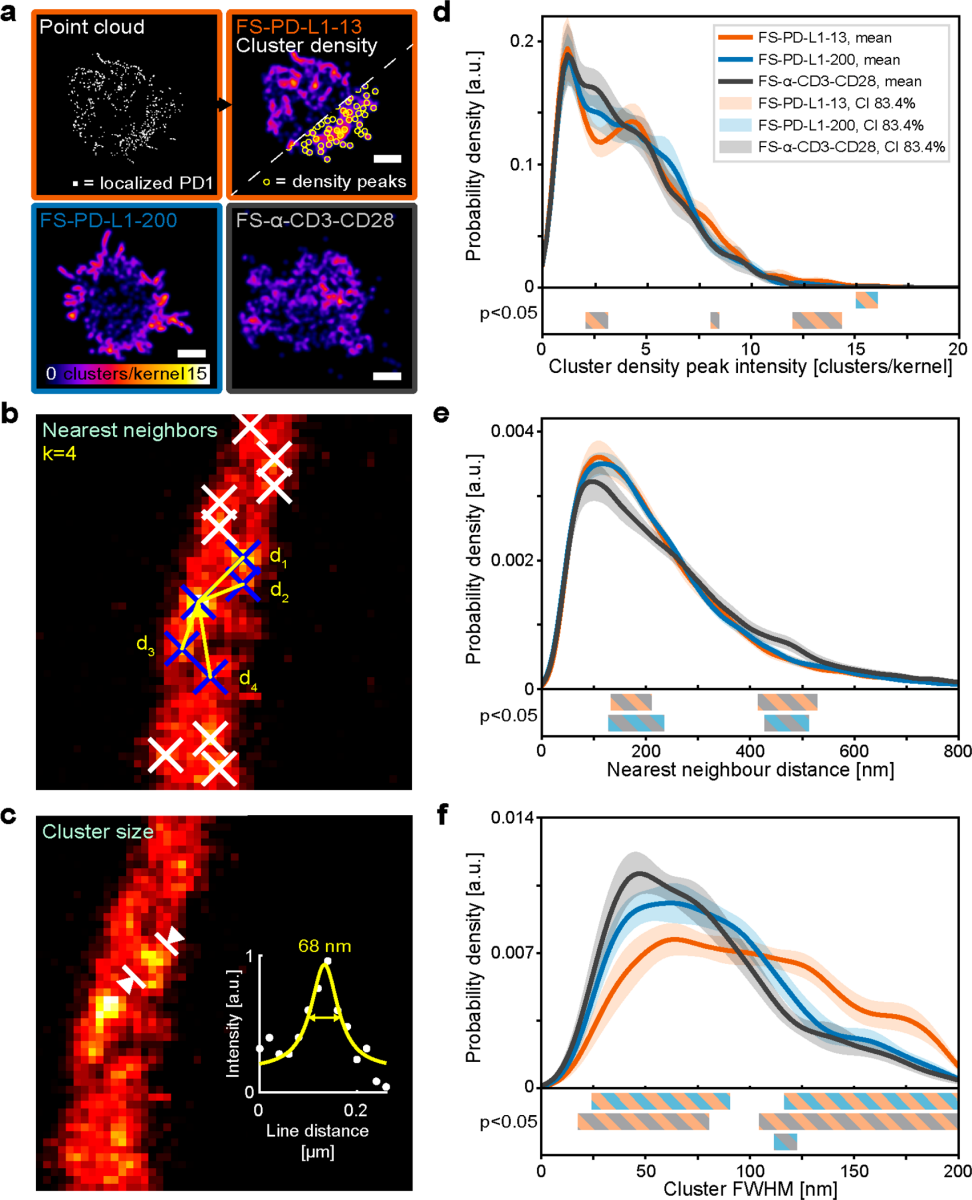
Analysis of PD-1 nanoclusters from STED microscopy data. (a) Point cloud and corresponding cluster density maps. Representative cluster density maps from each flat sheet condition, with intensity corresponding to the local density of localized clusters (number of clusters per Gaussian kernel with bandwidth = 200 nm). Scale bar = 3 μm. (b) Example image of *k*-nearest neighbor analysis for a cluster of interest (middle blue cross) with the four nearest neighbors (surrounding blue crosses) and the distances d_n_ between them (yellow lines). (c) Example image showing cluster size analysis of an individual cluster with a line profile marked and plotted (white circles), providing a full width at half-maximum (FWHM) of 68 nm from a Lorentzian fit to the data (yellow line). (d–f) Results from respective analysis; (d) peak intensity distributions from cluster density maps, (e) nearest neighbor distance distributions and (f) cluster size distributions shown as mean kernel density estimation (solid lines) of the resulting distribution from each cell (*N* = 24). Corresponding 83.4% confidence intervals of the mean are indicated by shaded areas per condition, FS-PD-L1–13 (orange), FS-PD-L1–200 (blue) and FS-α CD3-CD28 (gray). Regions of significant difference between conditions are marked by colored bars (bottom, FS-PD-L1–13/FS-α-CD3-CD28 (orange/gray), FS-PD-L1–200/FS-α-CD3-CD28 (blue/gray) and FS-PD-L1-13/FS-PD-L1-200 (orange/blue)). *N* = 24 cells for each flat sheet condition from 2 glasses from 2 days of experiment (N_1_ = 15, N_2_ = 9 for each condition), a total of 72 cells were analyzed (24 cells × 3 conditions).

First, we generated cluster density maps through kernel density estimation of the cluster localization point cloud (Figure 5a). By analyzing the local cluster densities, we observed that both FS-PD-L1-13 and FS-PD-L1-200 induced more areas with higher density of PD-1 clusters as compared to a more evenly spread PD-1 distribution in FS-α-CD3-CD28 without PD-L1 stimulation (Figure 5d and Figure S24). Despite displaying a high cell-to-cell variability in the cluster density maps (Figure S22), FS-PD-L1-13 on average resulted in more areas with higher PD-1 density (one-sided Kolmogorov–Smirnov test: FS-PD-L1-13/FS-α-CD3-CD28: *P* < 0.0001, FS-PD-L1-13/FS-PD-L1–200: *P* < 0.001) (Figure S24).

We next analyzed the nearest neighbor distances between the PD-1 cluster localizations (Figure 5b and 5e). FS-PD-L1-13 and FS-PD-L1-200 both resulted in distributions of PD-1 clusters that were shifted toward shorter distances as compared to FS-α-CD3-CD28, with ranges of significant difference (nonoverlapping confidence intervals) at 134–211 and 131–233 nm, respectively (Figure 5e). The largest differences in mean probability density appeared at 160 nm (FS-PD-L1-13) and 155 nm (FS-PD-L1-200). Because of the increased probability at shorter distances and the fixed *k* in the analysis, we additionally saw a significant decrease in the mean probability density distributions of FS-PD-L1-13 and FS-PD-L1-200 as compared to FS-α-CD3-CD28 at 412–532 and 431–511 nm, respectively. Moreover, the maximum cumulative difference in the number of nearest neighbor distances between FS-PD-L1-200 and FS-α-CD3-CD28 was found at the crossover point of the two probability density functions at 264 nm, indicating more neighbors were within 264 nm for FS-PD-L1-200. For FS-PD-L1-13 compared to FS-α-CD3-CD28, the maximum cumulative difference was found at 237 nm. Overall, these results indicate that there is a significant effect on nearest neighbor distances of PD-1 clusters induced by FS-PD-L1-200 and FS-PD-L1-13.

Finally, FS-PD-L1-13 resulted in significant differences in PD-1 cluster sizes as compared to FS-α-CD3-CD28, with a clear distribution shift toward larger cluster sizes (>100 nm) (Figure 5c,f). In contrast, FS-PD-L1-200 induced a cluster size distribution more like FS-α-CD3-CD28, and the two conditions were more likely to form smaller PD-1 cluster sizes (<100 nm) than FS-PD-L1-13. A small subpopulation of larger PD-1 nanoclusters was also observed in FS-PD-L1-200, which significantly differed with FS-α-CD3-CD28 at 111–123 nm (Figure 5f). The maximum cumulative differences (crossover points) indicate that FS-PD-L1-200 was more likely to result in PD-1 cluster sizes above 76 nm than FS-α-CD3-CD28, while FS-PD-L1-13 showed a maximum difference at 94 and 103 nm as compared to FS-α-CD3-CD28 and FS-PD-L1-200, respectively.

Overall, FS-PD-L1-13 and FS-PD-L1-200 resulted in areas of dense PD-1 clusters of similar cluster–cluster distances. FS-PD-L1-200 mostly induced formation of smaller PD-1 nanoclusters (50–100 nm), while FS-PD-L1-13 mostly generated larger PD-1 nanoclusters (>100 nm). The latter could be triggered by the 13 nm PD-L1 interligand distance on the flat sheets bringing two PD-1 receptors in proximity, which is inseparable by STED imaging. Interestingly, FS-PD-L1-200 also triggered a small subset of large PD-1 nanoclusters (∼120 nm) which could be caused by the ends of flat sheets meeting each other, thus bringing multiple PD-L1 proteins in proximity.

Together, our results indicate that the nanoscale spatial organization of PD-L1 regulates PD-1-mediated inhibition of T-cell activation. Interestingly, the nonoverlapping surface distributions of antibody- and PD-L1-flat sheets (Figure S21) imply a separation between PD-L1 proteins in FS-PD-L1-200 and CD3/CD28 activating antibodies in FS-α-CD3-CD28 of at least 50 nm. This suggests that proximity between ligand bound PD-1 and activating antibody bound-TCR and CD28 is not a requirement for PD-L1-mediated inhibition of T-cell activation in this system. Further, we demonstrated that FS-PD-L1, FS-PD-L1-13, and FS-PD-L1-40 presented PD-L1 proteins to T cells at fixed positions that were not conducive to inhibition of T-cell activation. This suggests that the nanoscale organization of PD-L1 proteins can be exploited to prevent inhibition of T-cell activation, in a manner that is orthogonal to PD-1 blockade using anti-PD-1 antibodies.

## Conclusions

In this study, we used wireframe DNA origami as a tool to investigate the effects of the nanoscale spatial organization of PD-L1 on T-cell activation. We demonstrated that DNA origami flat sheets with a single PD-L1 protein or two PD-L1 proteins separated by 13 or 40 nm did not cause inhibition of CD3/CD28-mediated T-cell signaling. In contrast, DNA origami flat sheets with two PD-L1 proteins separated by 200 nm, and presented at the edges of the nanostructures, suppressed T-cell signaling. Overall, our results provide insights into the spatial relationships between PD-1/PD-L1 immune checkpoints and T-cell signaling. These findings can provide a foundation for rationally designed nanotherapeutic-based platforms in cancer immunotherapy.^49,50^

## Methods

### Antibody Conjugation

Mouse anti-human CD3ε IgG2a (OKT3, eBioscience) and mouse anti-human CD28 IgG1 (CD28.2, eBioscience) at a concentration of 1 mg/mL were buffer-exchanged to 1× PBS, pH 7.4 using Zeba Spin Desalting columns, 7K MWCO (ThermoFisher Scientific). DBCO-NHS reagent (Click Chemistry Tools) was resuspended in *N*,*N*-dimethylformamide at 10 mM concentration. This was added to the antibody mixes at a molar ratio of 10:1 and incubated on ice for 2 h. The reaction was quenched with 75 mM Tris–HCl, pH 8 on ice for 15 min. Nonreactive DBCO-NHS esters were removed by buffer-exchange to 1× PBS, pH 7.4 using Zeba Spin Desalting columns, 7K MWCO. Azide-modified oligos, Tag 1 and Tag 2 (Table S1) were added to anti-CD3ε IgG2a and CD28 IgG1, respectively, at a molar ratio of 2:1 based on the molarity of DBCO incorporated. The reactions were then incubated overnight at 4 °C. The conjugates were added into Amicon Ultra-0.5 centrifugal filter units with Ultracel-50 membrane (Merck Millipore) coated with 5% Pluronic-F127 (w/v) (ThermoFisher Scientific) and washed 8 times with 1 × PBS, pH 7.4 (centrifugation 14,000× g, 2 min) to remove unconjugated oligos. After washing, antibody–oligo conjugates were concentrated to about 100 μL volume. Antibody–oligo conjugate concentrations were determined using the Micro BCA Protein Assay kit (ThermoFisher Scientific) according to manufacturer’s instructions.

### PD-L1 Conjugation

Recombinant human PD-L1 His-tag (9049-B7-100, R&D Systems) was reconstituted with 1 × PBS, pH 6.3 according to manufacturer’s guidelines. The protein was conjugated to an azide-modified oligo, Tag 3 (Table S1) using *bis*-sulfone-PEG_4_-DBCO compound (Click Chemistry Tools) as previously described,^33,45^ with a molar ratio of 1:2 (molarity of DBCO incorporated: molarity of oligo). Protein–oligo conjugates were purified using an Amicon Ultra-0.5 centrifugal filter unit with Ultracel-30 membrane (Merck Millipore) coated with 5% Pluronic-F127 (w/v) (ThermoFisher Scientific).

Validation of antibody–oligo conjugates and PD-L1–oligo conjugates was performed by hybridizing complementary Alexa Fluor-labeled oligos (Supporting Information Table S2) for 1 h at 37 °C as previously described. Antibody–oligo conjugates (1.3 μM) were mixed with complementary Alexa Fluor 647–oligos (4 μM) and PD-L1–oligo conjugates (5 μM) were mixed with Alexa Fluor 488–oligos (2 μM) and the mixes were run on a native 4–20% Mini-Protean TGX Precast gel (Bio-Rad) in 1 × Tris-glycine buffer at 200 V for 34 min. The gels were imaged with ImageQuant LAS 4010 system (GE Healthcare) with fluorescence transillumination and then stained using Pierce Silver Stain kit (ThermoFisher Scientific).

### Protein Flat Sheet Production

Staple strand sequences for folding the 3-tessellation flat sheet were described previously.^43^ The 216 core staples were obtained from Integrated DNA Technologies at a concentration of 100 μM in water in three 96-well plates. Staple mixes for folding each flat sheet design were prepared to a final concentration of 463 nM based on the pipetting schemes outlined in Supporting Information Figure S25 with some core staples replaced by protruding/modified staples (Tables S3 and S4). The flat sheets were produced as previously described with the following minor modifications.^43^ In the folding reactions, the p8064 scaffold (Tilibit) was mixed at concentration of 20 with 200 nM of staples in 1 × PBS, pH 7.4. After removal of excess staples with 100 kDa MWCO Amicon centrifugal filters (Milipore), the concentration of flat sheets was adjusted to a concentration of 20 nM in 1 × PBS, pH 7.4. Hybridization of protein–oligo conjugates was performed as demonstrated previously,^32^ in which protein–oligo conjugates were added at 5-fold (one site) or 10-fold (two sites) molar excess of the flat sheets. Excess protein–oligo conjugates were removed passing the samples consecutively through three spin columns packed with 400 μL of Sepharose 6B resin *via* centrifugation at 800*g*, for 3 min.

### AFM Imaging

After Sepharose purification, protein–DNA flat sheets were imaged on a disc of mica fastened with epoxy adhesive to the center of a microscope slide and enclosed by a plastic ring attached to the slide using repro rubber. The flat sheets were diluted to 1 nM in TE-Mg buffer (5 mM Tris base, 1 mM EDTA, 10 mM MgCl_2_, pH 8.0), and 10 μL was pipetted onto freshly cleaved mica. After 30 s, 4 μL of 5 mM NiSO_4_ was added and incubated for a further 4.5 min. The surface was then rinsed with 1 mL of 0.1 μm-filtered TE-Mg buffer after which 1.5 mL of filtered TE-Mg buffer was added to the mica disk for imaging. Imaging was performed in liquid using a JPK Instruments NanoWizard 3 Ultra AFM with a Bruker AC40 cantilever in AC mode.

### Immunolabeling and Agarose Gel Electrophoresis for Characterization of Protein Flat Sheets

Flat sheets hybridized with anti-CD3 IgG and anti-CD28 IgG (4 μL volume) were mixed with 40 nM Alexa Fluor 647-conjugated AffiniPure rabbit anti-mouse IgG, Fcγ fragment specific (no. 315-605-008, Jackson ImmunoResearch). Similarly, flat sheets hybridized with PD-L1 were mixed with Alexa Fluor 488-conjugated anti-PD-L1 antibody (R&D Systems). The mixes were incubated for 15 min at room temperature before being loaded onto 2% agarose gels (0.5× TBE, 11 mM MgCl_2_ and 0.5 mg/mL ethidium bromide) and ran at 90 V for 3 h in an ice bath. The gel was imaged in the ImageQuant LAS 4010 system (GE Healthcare), using UV, Cy5 and SYBR Green transillumination. The final concentration of the protein–DNA flat sheets was determined by the ratio of gel band intensity before and after Sepharose purification and calculated using the concentration of flat sheets prior purification as a reference.

### Cell Culture

Jurkat E6-1 (ATCC), PD-1-NFAT luciferase reporter Jurkat (BPS Bioscience) and PD-1-SNAP-Jurkat cells were maintained in complete RPMI 1640 supplemented with 10% heat-inactivated fetal bovine serum (ThermoFisher Scientific). The cell lines were routinely screened for mycoplasma contamination (Eurofins Genomics).

### Luciferase Reporter Assay

For surface immobilization of protein flat sheets, 10 μg/mL of biotinylated-BSA (29130, ThermoFisher Scientific) was coated in a 96-well plate for 30 min at 37 °C. The wells were washed five times with 1 × PBS, pH 7.4 and then coated with 10 μg/mL streptavidin (21122, ThermoFisher Scientific) for 30 min at 37 °C. The wells were first coated with either FS-empty (10.5 nM), FS-α-CD3 (0.5 nM), or FS- FS-α-CD3-CD28 (0.5 nM) in 1× PBS, pH 7.4 for 30 min at 37 °C. After washing five times with PBS, FS-α-CD3 or FS-α-CD3-CD28-coated wells were either coated with FS-empty (10 nM), FS-PD-L1 (10 nM), FS-PD-L1-13 (5 nM), FS-PD-L1-40 (5 nM), or FS-PD-L1-200 (5 nM) for 30 min at 37 °C. After incubation, the wells were subjected to five washes of PBS. Wells precoated with 5 nM of FS-PD-L1-13, FS-PD-L1-40, and FS-PD-L1-200 were treated with another coating of 5 nM FS-empty, to ensure all wells had a similar level of flat sheet coating. After washing with PBS, PD-1-NFAT cells were seeded at 3 × 10^4^ cells per well and incubated for 3 h at 37 °C, 5% CO_2_. Cells were lysed with 1 × Glo Lysis buffer (Promega), and luciferase reporter activity was measured using Bright-Glo Luciferase Assay System (Promega) in a Varioskan LUX multimode microplate reader.

### Statistical Analysis

Statistical significances for Figure 3, Supporting Information Figure S18, S19c and S20 were evaluated by one-way ANOVA and Tukey multiple pairwise comparison with *R* package *ggplot2*.^51^ The number of biological repeats was N = 3, unless otherwise stated. Shapiro-Wilk and Levene’s tests were employed to check for violations of normality and homogeneity of variances. Statistical significance for the mean cell distributions in Figure 5d-f was evaluated comparing the confidence intervals, extracted as explained under “STED image pre-processing and cluster localization”. Nonoverlapping confidence intervals correspond to a significant difference in a 95% Student’s *t* test (*p* < 0.05), while overlapping confidence intervals similarly correspond to no significant difference. Statistical significance for the pooled distributions in Figure S24, based on data presented in Figure 5d in the Results section, was evaluated with one-sided two-sample Kolmogorov–Smirnov tests, testing the hypothesis that distribution A is bigger than distribution B.

### Sample Preparation for STED Microscopy

Unless otherwise stated, incubations were performed at room temperature. Round cover glasses (18 mm, 1.5H) were cleaned with acetone and isopropanol and dried with N_2_ gas. Each cover glass was placed on a parafilm containing a drop (120 μL) of 1 mg/mL biotinylated-BSA (ThermoFisher Scientific) for 5 min to allow adsorption to the glass surface. Cover glasses were then washed twice in 1 × PBS and coated with 0.5 mg/mL streptavidin (ThermoFisher Scientific) in the same manner. After washing twice in 1 × PBS, each cover glass was incubated with a drop of FS-α-CD3-CD28 (0.5 nM) for 10 min. The coated cover glasses were washed twice and then incubated with 5 nM of either FS-empty, FS-PD-L1–13 or FS-PD-L1–200. PD-1-SNAP cells were labeled with 5 μM SNAP-Cell-647-SiR (New England Biolabs) according to manufacturer’s instructions at 37 °C for 30 min with gentle mixing at 300 rpm. The labeled cells were stimulated on the flat sheet-coated coverslips in a 12 -well plate for 10 min and then fixed with 4% paraformaldehyde in 1 × PBS for 20 min. The cover glasses were carefully washed three times in PBS before mounting in Mowiol mounting media.

### STED Microscopy Setup and Imaging

The STED microscopy imaging was performed with a custom-built STED setup operating in the far-red wavelength regime as previously described.^52^ The 647-SiR dye was excited with a 640 nm pulsed diode laser (LDH-D-C-640, PicoQuant) and subsequently depleted with a 775 nm pulsed laser (KATANA 08 HP, OneFive), both operating at 40 MHz. A HC PL APO 100*x*/1.40 Oil STED White objective lens (15506378, Leica Microsystems) was used, through which the fluorescence signal was collected and detected through a bandpass filter (GT670/40m, Chroma Technology) and a notch filter (NF03–785E-25, Semrock) with a free-space APD (SPCM-AQRH-13-TR, Excelitas Technologies). The setup includes a focus lock to ensure a constant focal plane is held throughout the image. The setup is controlled with Tempesta, a custom-written open-source microscope control software in Python (https://github.com/jonatanalvelid/Tempesta-RedSTED).

The STED imaging was done with a 640 nm excitation laser power of 10.3–12.8 μW and a 775 nm depletion laser power of 76.2 mW, both measured at the first conjugate back focal plane of the objective. The pixel size for all STED images was set to 20–25 nm. Each image was acquired by adding up 4-line scans for each line, each with a pixel dwell time of 15–20 μs, resulting in a total pixel dwell time of 60–80 μs. The focus lock was used, ensuring imaging of the surface-contacting cell membrane plane of the cells. All STED images shown and used for image analysis were raw data. To aid visualization, we applied a Gaussian smoothing with 10 nm radius in STED images.

### STED Image Preprocessing and Cluster Localization

The image analysis on the PD-1 receptor STED image data was performed through a custom-written pipeline in Python (https://github.com/jonatanalvelid/ImmunocellReceptorAnalysis). First, common preprocessing and cluster localization were performed. The cluster coordinates were subsequently used to analyze (1) the local cluster density throughout the cell through a kernel density estimate (KDE) map, (2) the nearest neighbor distances for each cluster through a *k*-nearest neighbor algorithm, and (3) the cluster sizes through line profile tracing and fitting with Lorentzian functions.

The image preprocessing was performed by starting with a binarization of the raw image. The out-of-the-cell background was removed through a multiplication of the raw image with the binary cell map. Second, a difference of Gaussians filter (σ1 = 0 nm, σ2 = 100 nm) was performed using the implementation in the scikit-image package.^53^ Any negative values were changed to 0, and a Gaussian smoothing with σ = 15 nm was performed. To ensure similar intensity ranges in data sets with varied acquisition settings, an image standardization step was performed by diving pixel counts with the sum of the mean and standard deviation of the cell. Finally, clusters were detected by using the local peak detection implementation from the scikit-image package. To minimize false positive peak detections, a global peak intensity threshold was used and separately set for each day of the experiment. Detected peaks in adjacent pixels were merged to avoid multiple localizations from the same cluster.

### Cluster Analysis

Local cluster density analysis was performed by fitting 2D KDE maps from the point clouds of cluster localizations, using the implementation in scikit-learn package.^54^ We used a Gaussian kernel with a bandwidth of 200 nm. The shown intensity is the kernel-weighted number of clusters present around each pixel. The 2D KDE maps were then analyzed by detecting the peaks using the local peak detection from the scikit-image package. The intensity from each peak was then extracted, building up a distribution of density peak intensities for each cell.

Nearest neighbor analysis was performed as a *k*-nearest neighbor analysis, with *k* = 4 (excluding self), using the implementation in scikit-learn package. For each cell, a distribution containing four nearest neighbor distances per cluster was extracted. *k* = 4 was chosen to ensure the inclusion of nearly all clusters inside a radius of ∼200 nm from each cluster, while excluding nearly all clusters further away than ∼800 nm.

Cluster size analysis was performed through line profile fitting of four 400 nm long line profiles (0°, 45°, 90°, 135°) per localized cluster. The full width at half-maximum (FWHM) was extracted from a Lorentzian fit if the R^2^ value of the fitting was higher than a threshold (0.9). The minimum extracted value was taken as the cluster FWHM. For each cell, a distribution containing these cluster FWHM values was extracted.

The results from each of the three analyses were presented as the mean kernel density estimation of the individual kernel density estimations of the distributions from each cell. The 83.4% confidence interval for the mean was calculated using the implementation in the scipy package.^55^ Graphs were prepared using the matplotlib package.^56^

## Supporting Information Available

Additional methods, DNA sequences, quantifications of native-PAGE images, reducing SDS-PAGE, vHelix design of DNA flat sheet nanostructures, AFM images and their quantification, SPR analysis, agarose gel images, NFAT-luciferase experiments, flow cytometry, Western blots, IL-2 Elisa, IL-2 qPCR, confocal microscopy images, STED images, and pipetting schemes (PDF)

## References

[ref1] Nguyen L. T.; Ohashi P. S. Clinical Blockade of PD1 and LAG3-Potential Mechanisms of Action. Nat. Rev. Immunol. 2015, 15, 45–56. 10.1038/nri3790. 25534622

[ref2] Sharpe A. H.; Pauken K. E. The Diverse Functions of the PD1 Inhibitory Pathway. Nat. Rev. Immunol. 2018, 18, 153–167. 10.1038/nri.2017.108. 28990585

[ref3] Agata Y.; Kawasaki A.; Nishimura H.; Ishida Y.; Tsubata T.; Yagita H.; Honjo T. Expression of the PD-1 Antigen on the Surface of Stimulated Mouse T and B Lymphocytes. Int. Immunol. 1996, 8, 765–772. 10.1093/intimm/8.5.765. 8671665

[ref4] Sheppard K. A.; Fitz L. J.; Lee J. M.; Benander C.; George J. A.; Wooters J.; Qiu Y.; Jussif J. M.; Carter L. L.; Wood C. R.; Chaudhary D. PD-1 Inhibits T-Cell Receptor Induced Phosphorylation of the ZAP70/CD3ζ Signalosome and Downstream Signaling to PKCθ. FEBS Lett. 2004, 574, 37–41. 10.1016/j.febslet.2004.07.083. 15358536

[ref5] Patsoukis N.; Brown J.; Petkova V.; Liu F.; Li L.; Boussiotis V. A. Selective Effects of PD-1 on Akt and Ras Pathways Regulate Molecular Components of the Cell Cycle and Inhibit T Cell Proliferation. Sci. Signaling 2012, 5, ra46 10.1126/scisignal.2002796. PMC5498435 22740686

[ref6] Mizuno R.; Sugiura D.; Shimizu K.; Maruhashi T.; Watada M.; Okazaki I.-M.; Okazaki T. PD-1 Primarily Targets TCR Signal in the Inhibition of Functional T Cell Activation. Front. Immunol. 2019, 10, 630 10.3389/fimmu.2019.00630. 31001256 PMC6455061

[ref7] Freeman G. J.; Long A. J.; Iwai Y.; Bourque K.; Chernova T.; Nishimura H.; Fitz L. J.; Malenkovich N.; Okazaki T.; Byrne M. C.; Horton H. F.; Fouser L.; Carter L.; Ling V.; Bowman M. R.; Carreno B. M.; Collins M.; Wood C. R.; Honjo T. Engagement of the PD-1 Immunoinhibitory Receptor by a Novel B7 Family Member Leads to Negative Regulation of Lymphocyte Activation. J. Exp. Med. 2000, 192, 1027–1034. 10.1084/jem.192.7.1027. 11015443 PMC2193311

[ref8] Latchman Y.; Wood C. R.; Chernova T.; Chaudhary D.; Borde M.; Chernova I.; Iwai Y.; Long A. J.; Brown J. A.; Nunes R.; Greenfield E. A.; Bourque K.; Boussiotis V. A.; Carter L. L.; Carreno B. M.; Malenkovich N.; Nishimura H.; Okazaki T.; Honjo T.; Sharpe A. H.; et al. PD-L2 Is a Second Ligand for PD-1 and Inhibits T Cell Activation. Nat. Immunol. 2001, 2, 261–268. 10.1038/85330. 11224527

[ref9] Curiel T. J.; Wei S.; Dong H.; Alvarez X.; Cheng P.; Mottram P.; Krzysiek R.; Knutson K. L.; Daniel B.; Zimmermann M. C.; David O.; Burow M.; Gordon A.; Dhurandhar N.; Myers L.; Berggren R.; Hemminki A.; Alvarez R. D.; Emilie D.; Curiel D. T.; et al. Blockade of B7-H1 Improves Myeloid Dendritic Cell-Mediated Antitumor Immunity. Nat. Med. 2003, 9, 562–567. 10.1038/nm863. 12704383

[ref10] Ribas A.; Hamid O.; Daud A.; Hodi F. S.; Wolchok J. D.; Kefford R.; Joshua A. M.; Patnaik A.; Hwu W. J.; Weber J. S.; Gangadhar T. C.; Hersey P.; Dronca R.; Joseph R. W.; Zarour H.; Chmielowski B.; Lawrence D. P.; Algazi A.; Rizvi N. A.; Hoffner B.; et al. Association of Pembrolizumab with Tumor Response and Survival among Patients with Advanced Melanoma. JAMA, J. Am. Med. Assoc. 2016, 315, 1600–1609. 10.1001/jama.2016.4059. 27092830

[ref11] Robert C.; Long G. V.; Brady B.; Dutriaux C.; Maio M.; Mortier L.; Hassel J. C.; Rutkowski P.; McNeil C.; Kalinka-Warzocha E.; Savage K. J.; Hernberg M. M.; Lebbé C.; Charles J.; Mihalcioiu C.; Chiarion-Sileni V.; Mauch C.; Cognetti F.; Arance A.; Schmidt H.; et al. Nivolumab in Previously Untreated Melanoma without BRAF Mutation. N. Engl. J. Med. 2015, 372, 320–330. 10.1056/NEJMoa1412082. 25399552

[ref12] Keir M. E.; Liang S. C.; Guleria I.; Latchman Y. E.; Qipo A.; Albacker L. A.; Koulmanda M.; Freeman G. J.; Sayegh M. H.; Sharpe A. H. Tissue Expression of PD-L1Mediates Peripheral T Cell Tolerance. J. Exp. Med. 2006, 203, 883–895. 10.1084/jem.20051776. 16606670 PMC2118286

[ref13] Nishimura H.; Nose M.; Hiai H.; Minato N.; Honjo T. Development of Lupus-Like Autoimmune Diseases by Disruption of the PD-1 Gene Encoding an ITIM Motif-Carrying Immunoreceptor. Immunity 1999, 11, 141–151. 10.1016/S1074-7613(00)80089-8. 10485649

[ref14] Nishimura H.; Okazaki T.; Tanaka Y.; Nakatani K.; Hara M.; Matsumori A.; Sasayama S.; Mizoguchi A.; Hiai H.; Minato N.; Honjo T. Autoimmune Dilated Cardiomyopathy in PD-1 Receptor-Deficient Mice. Science 2001, 291, 319–322. 10.1126/science.291.5502.319. 11209085

[ref15] Paz-Ares L.; Luft A.; Vicente D.; Tafreshi A.; Gümüş M.; Mazières J.; Hermes B.; Çay Şenler F.; Csőszi T.; Fülöp A.; Rodríguez-Cid J.; Wilson J.; Sugawara S.; Kato T.; Lee K. H.; Cheng Y.; Novello S.; Halmos B.; Li X.; Lubiniecki G. M.; et al. Pembrolizumab plus Chemotherapy for Squamous Non-Small-Cell Lung Cancer. N. Engl. J. Med. 2018, 379, 2040–2051. 10.1056/NEJMoa1810865. 30280635

[ref16] Robert C.; Schachter J.; Long G. V.; Arance A.; Grob J. J.; Mortier L.; Daud A.; Carlino M. S.; McNeil C.; Lotem M.; Larkin J.; Lorigan P.; Neyns B.; Blank C. U.; Hamid O.; Mateus C.; Shapira-Frommer R.; Kosh M.; Zhou H.; Ibrahim N.; et al. Pembrolizumab *versus* Ipilimumab in Advanced Melanoma. N. Engl. J. Med. 2015, 372, 2521–2532. 10.1056/NEJMoa1503093. 25891173

[ref17] Ferreira R. C.; Castro Dopico X.; Oliveira J. J.; Rainbow D. B.; Yang J. H.; Trzupek D.; Todd S. A.; McNeill M.; Steri M.; Orrù V.; Fiorillo E.; Crouch D. J. M.; Pekalski M. L.; Cucca F.; Tree T. I.; Vyse T. J.; Wicker L. S.; Todd J. A. Chronic Immune Activation in Systemic Lupus Erythematosus and the Autoimmune PTPN22 Trp620 Risk Allele Drive the Expansion of FOXP3+ Regulatory T Cells and PD-1 Expression. Front. Immunol. 2019, 10, 2606 10.3389/fimmu.2019.02606. 31781109 PMC6857542

[ref18] Yokosuka T.; Takamatsu M.; Kobayashi-Imanishi W.; Hashimoto-Tane A.; Azuma M.; Saito T. Programmed Cell Death 1 Forms Negative Costimulatory Microclusters That Directly Inhibit T Cell Receptor Signaling by Recruiting Phosphatase SHP2. J. Exp. Med. 2012, 209, 1201–1217. 10.1084/jem.20112741. 22641383 PMC3371732

[ref19] Hui E.; Cheung J.; Zhu J.; Su X.; Taylor M. J.; Wallweber H. A.; Sasmal D. K.; Huang J.; Kim J. M.; Mellman I.; Vale R. D. T Cell Costimulatory Receptor CD28 Is a Primary Target for PD-1-Mediated Inhibition. Science 2017, 355, 1428–1433. 10.1126/science.aaf1292. 28280247 PMC6286077

[ref20] Demetriou P.; Abu-Shah E.; Valvo S.; McCuaig S.; Mayya V.; Kvalvaag A.; Starkey T.; Korobchevskaya K.; Lee L. Y. W.; Friedrich M.; Mann E.; Kutuzov M. A.; Morotti M.; Wietek N.; Rada H.; Yusuf S.; Afrose J.; Siokis A.; Meyer-Hermann M.; et al. A Dynamic CD2-Rich Compartment at the Outer Edge of the Immunological Synapse Boosts and Integrates Signals. Nat. Immunol. 2020, 21, 1232–1243. 10.1038/s41590-020-0770-x. 32929275 PMC7611174

[ref21] Zhao Y.; Harrison D. L.; Song Y.; Ji J.; Huang J.; Hui E. Antigen-Presenting Cell-Intrinsic PD-1 Neutralizes PD-L1 in Cis to Attenuate PD-1 Signaling in T Cells. Cell Rep. 2018, 24, 379–390. 10.1016/j.celrep.2018.06.054. 29996099 PMC6093302

[ref22] Okazaki T.; Maeda A.; Nishimura H.; Kurosaki T.; Honjo T. PD-1 Immunoreceptor Inhibits B Cell Receptor-Mediated Signaling by Recruiting Src Homology 2-Domain-Containing Tyrosine Phosphatase 2 to Phosphotyrosine. Proc. Natl. Acad. Sci. U. S. A. 2001, 98, 13866–13871. 10.1073/pnas.231486598. 11698646 PMC61133

[ref23] Chemnitz J. M.; Parry R. V.; Nichols K. E.; June C. H.; Riley J. L. SHP-1 and SHP-2 Associate with Immunoreceptor Tyrosine-Based Switch Motif of Programmed Death 1 upon Primary Human T Cell Stimulation, But Only Receptor Ligation Prevents T Cell Activation. J. Immunol. 2004, 173, 945–954. 10.4049/jimmunol.173.2.945. 15240681

[ref24] Kamphorst A. O.; Wieland A.; Nasti T.; Yang S.; Zhang R.; Barber D. L.; Konieczny B. T.; Daugherty C. Z.; Koenig L.; Yu K.; Sica G. L.; Sharpe A. H.; Freeman G. J.; Blazar B. R.; Turka L. A.; Owonikoko T. K.; Pillai R. N.; Ramalingam S. S.; Araki K.; Ahmed R. Rescue of Exhausted CD8 T Cells by PD-1-Targeted Therapies Is CD28-Dependent. Science 2017, 355, 1423–1427. 10.1126/science.aaf0683. 28280249 PMC5595217

[ref25] Celis-Gutierrez J.; Blattmann P.; Zhai Y.; Jarmuzynski N.; Ruminski K.; Grégoire C.; Ounoughene Y.; Fiore F.; Aebersold R.; Roncagalli R.; Gstaiger M.; Malissen B. Quantitative Interactomics in Primary T Cells Provides a Rationale for Concomitant PD-1 and BTLA Coinhibitor Blockade in Cancer Immunotherapy. Cell Rep. 2019, 27, 3315–3330. 10.1016/j.celrep.2019.05.041. 31189114 PMC6581740

[ref26] Zak K. M.; Grudnik P.; Guzik K.; Zieba B. J.; Musielak B.; Dömling A.; Dubin G.; Holak T. A. Structural Basis for Small Molecule Targeting of the Programmed Death Ligand 1 (PD-L1). Oncotarget 2016, 7, 30323–30335. 10.18632/oncotarget.8730. 27083005 PMC5058683

[ref27] Guzik K.; Zak K. M.; Grudnik P.; Magiera K.; Musielak B.; Törner R.; Skalniak L.; Dömling A.; Dubin G.; Holak T. A. Small-Molecule Inhibitors of the Programmed Cell Death-1/Programmed Death-Ligand 1 (PD-1/PD-L1) Interaction *via* Transiently Induced Protein States and Dimerization of PD-L1. J. Med. Chem. 2017, 60, 5857–5867. 10.1021/acs.jmedchem.7b00293. 28613862

[ref28] Skalniak L.; Zak K. M.; Guzik K.; Magiera K.; Musielak B.; Pachota M.; Szelazek B.; Kocik J.; Grudnik P.; Tomala M.; Krzanik S.; Pyrc K.; Dömling A.; Dubin G.; Holak T. A. Small-Molecule Inhibitors of PD-1/PD-L1 Immune Checkpoint Alleviate the PD-L1-Induced Exhaustion of T-Cells. Oncotarget 2017, 8, 72167–72181. 10.18632/oncotarget.20050. 29069777 PMC5641120

[ref29] Mahoney K. M.; Shukla S. A.; Patsoukis N.; Chaudhri A.; Browne E. P.; Arazi A.; Eisenhaure T. M.; Pendergraft W. F.; Hua P.; Pham H. C.; Bu X.; Zhu B.; Hacohen N.; Fritsch E. F.; Boussiotis V. A.; Wu C. J.; Freeman G. J. A Secreted PD-L1 Splice Variant That Covalently Dimerizes and Mediates Immunosuppression. Cancer Immunol. Immunother. 2019, 68, 421–432. 10.1007/s00262-018-2282-1. 30564891 PMC6426808

[ref30] Voigt N. V.; Tørring T.; Rotaru A.; Jacobsen M. F.; Ravnsbæk J. B.; Subramani R.; Mamdouh W.; Kjems J.; Mokhir A.; Besenbacher F.; Gothelf K. V. Single-Molecule Chemical Reactions on DNA Origami. Nat. Nanotechnol. 2010, 5, 200–203. 10.1038/nnano.2010.5. 20190747

[ref31] Rinker S.; Ke Y.; Liu Y.; Chhabra R.; Yan H. Self-Assembled DNA Nanostructures for Distance-Dependent Multivalent Ligand-Protein Binding. Nat. Nanotechnol. 2008, 3, 418–422. 10.1038/nnano.2008.164. 18654566 PMC2556356

[ref32] Shaw A.; Lundin V.; Petrova E.; Fördos F.; Benson E.; Al-Amin A.; Herland A.; Blokzijl A.; Högberg B.; Teixeira A. I. Spatial Control of Membrane Receptor Function Using Ligand Nanocalipers. Nat. Methods 2014, 11, 841–846. 10.1038/nmeth.3025. 24997862

[ref33] Verheyen T.; Fang T.; Lindenhofer D.; Wang Y.; Akopyan K.; Lindqvist A.; Högberg B.; Teixeira A. I. Spatial Organization-Dependent EphA2 Transcriptional Responses Revealed by Ligand Nanocalipers. Nucleic Acids Res. 2020, 48, 4777–5787. 10.1093/nar/gkaa274. PMC7261182 32352518

[ref34] Huang D.; Patel K.; Perez-Garrido S.; Marshall J. F.; Palma M. DNA Origami Nanoarrays for Multivalent Investigations of Cancer Cell Spreading with Nanoscale Spatial Resolution and Single-Molecule Control. ACS Nano 2019, 13, 728–736. 10.1021/acsnano.8b08010. 30588806

[ref35] Veneziano R.; Moyer T. J.; Stone M. B.; Wamhoff E. C.; Read B. J.; Mukherjee S.; Shepherd T. R.; Das J.; Schief W. R.; Irvine D. J.; Bathe M. Role of Nanoscale Antigen Organization on B-Cell Activation Probed Using DNA Origami. Nat. Nanotechnol. 2020, 15, 716–723. 10.1038/s41565-020-0719-0. 32601450 PMC7415668

[ref36] Delcassian D.; Depoil D.; Rudnicka D.; Liu M.; Davis D. M.; Dustin M. L.; Dunlop I. E. Nanoscale Ligand Spacing Influences Receptor Triggering in T Cells and NK Cells. Nano Lett. 2013, 13, 5608–5614. 10.1021/nl403252x. 24125583 PMC4288448

[ref37] Meddens M. B. M.; Mennens S. F. B.; Celikkol F. B.; Te Riet J.; Kanger J. S.; Joosten B.; Witsenburg J. J.; Brock R.; Figdor C. G.; Cambi A. Biophysical Characterization of CD6 - TCR/CD3 Interplay in T Cells. Front. Immunol. 2018, 9, 2333 10.3389/fimmu.2018.02333. 30356797 PMC6189472

[ref38] Doh J.; Irvine D. J. Immunological Synapse Arrays: Patterned Protein Surfaces That Modulate Immunological Synapse Structure Formation in T Cells. Proc. Natl. Acad. Sci. U. S. A. 2006, 103, 5700–5705. 10.1073/pnas.0509404103. 16585528 PMC1458636

[ref39] Mayya V.; Judokusumo E.; Abu Shah E.; Peel C. G.; Neiswanger W.; Depoil D.; Blair D. A.; Wiggins C. H.; Kam L. C.; Dustin M. L. Durable Interactions of T Cells with T Cell Receptor Stimuli in the Absence of a Stable Immunological Synapse. Cell Rep. 2018, 22, 340–349. 10.1016/j.celrep.2017.12.052. 29320731 PMC5775504

[ref40] Bashour K. T.; Gondarenko A.; Chen H.; Shen K.; Liu X.; Huse M.; Hone J. C.; Kam L. C. CD28 and CD3 Have Complementary Roles in T-Cell Traction Forces. Proc. Natl. Acad. Sci. U. S. A. 2014, 111, 2241–2246. 10.1073/pnas.1315606111. 24469820 PMC3926067

[ref41] Lambert L. H.; Goebrecht G. K. E.; De Leo S. E.; O’Connor R. S.; Nunez-Cruz S.; Li T.; De Yuan J.; Milone M. C.; Kam L. C. Improving T Cell Expansion with a Soft Touch. Nano Lett. 2017, 17, 821–826. 10.1021/acs.nanolett.6b04071. 28122453 PMC5504474

[ref42] Benson E.; Mohammed A.; Gardell J.; Masich S.; Czeizler E.; Orponen P.; Högberg B. DNA Rendering of Polyhedral Meshes at the Nanoscale. Nature 2015, 523, 441–444. 10.1038/nature14586. 26201596

[ref43] Benson E.; Mohammed A.; Bosco A.; Teixeira A. I.; Orponen P.; Högberg B. Computer-Aided Production of Scaffolded DNA Nanostructures from Flat Sheet Meshes. Angew. Chem., Int. Ed. 2016, 55, 8869–8872. 10.1002/anie.201602446. PMC6680348 27304204

[ref44] Macian F. NFAT Proteins: Key Regulators of T-Cell Development and Function. Nat. Rev. Immunol. 2005, 5, 472–484. 10.1038/nri1632. 15928679

[ref45] Cong Y.; Pawlisz E.; Bryant P.; Balan S.; Laurine E.; Tommasi R.; Singh R.; Dubey S.; Peciak K.; Bird M.; Sivasankar A.; Swierkosz J.; Muroni M.; Heidelberger S.; Farys M.; Khayrzad F.; Edwards J.; Badescu G.; Hodgson I.; Heise C.; et al. Site-Specific PEGylation at Histidine Tags. Bioconjugate Chem. 2012, 23, 248–263. 10.1021/bc200530x. 22243664

[ref46] Lillemeier B. F.; Mörtelmaier M. A.; Forstner M. B.; Huppa J. B.; Groves J. T.; Davis M. M. TCR and Lat Are Expressed on Separate Protein Islands on T Cell Membranes and Concatenate during Activation. Nat. Immunol. 2010, 11, 90–96. 10.1038/ni.1832. 20010844 PMC3273422

[ref47] Negulescu P. A.; Shastri N.; Cahalan M. D. Intracellular Calcium Dependence of Gene Expression in Single T Lymphocytes. Proc. Natl. Acad. Sci. U. S. A. 1994, 91, 2873–2877. 10.1073/pnas.91.7.2873. 8146203 PMC43473

[ref48] Jutz S.; Leitner J.; Schmetterer K.; Doel-Perez I.; Majdic O.; Grabmeier-Pfistershammer K.; Paster W.; Huppa J. B.; Steinberger P. Assessment of Costimulation and Coinhibition in a Triple Parameter T Cell Reporter Line: Simultaneous Measurement of NF-KB, NFAT and AP-1. J. Immunol. Methods 2016, 430, 10–20. 10.1016/j.jim.2016.01.007. 26780292

[ref49] Perica K.; Tu A.; Richter A.; Bieler J. G.; Edidin M.; Schneck J. P. Magnetic Field-Induced T Cell Receptor Clustering by Nanoparticles Enhances T Cell Activation and Stimulates Antitumor Activity. ACS Nano 2014, 8, 2252–2260. 10.1021/nn405520d. 24564881 PMC4004316

[ref50] Fadel T. R.; Sharp F. A.; Vudattu N.; Ragheb R.; Garyu J.; Kim D.; Hong E.; Li N.; Haller G. L.; Pfefferle L. D.; Justesen S.; Harold K. C.; Fahmy T. M. A Carbon Nanotube-Polymer Composite for T-Cell Therapy. Nat. Nanotechnol. 2014, 9, 639–647. 10.1038/nnano.2014.154. 25086604

[ref51] Wickham H. Ggplot2: Elegant Graphics for Data Analysis; Springer International Publishing AG Switzerland, 2016; pp 3–253.

[ref52] Alvelid J.; Testa I. Stable Stimulated Emission Depletion Imaging of Extended Sample Regions. J. Phys. D: Appl. Phys. 2020, 53, 024001 10.1088/1361-6463/ab4c13.

[ref53] Van Der Walt S.; Schönberger J. L.; Nunez-Iglesias J.; Boulogne F.; Warner J. D.; Yager N.; Gouillart E.; Yu T. Scikit-Image: Image Processing in Python. PeerJ 2014, 2, e453 10.7717/peerj.453. 25024921 PMC4081273

[ref54] Pedregosa F.; Varoquaux G.; Gramfort A.; Michel V.; Thirion B.; Grisel O.; Blondel M.; Prettenhofer P.; Weiss R.; Dubourg V.; Vanderplas J.; Passos A.; Cournapeau D.; Brucher M.; Perrot M.; Duchesnay É. Scikit-Learn: Machine Learning in Python. J. Mach. Learn. Res. 2011, 12, 2825–2830.

[ref55] Virtanen P.; Gommers R.; Oliphant T. E.; Haberland M.; Reddy T.; Cournapeau D.; Burovski E.; Peterson P.; Weckesser W.; Bright J.; van der Walt S. J.; Brett M.; Wilson J.; Millman K. J.; Mayorov N.; Nelson A. R. J.; Jones E.; Kern R.; Larson E.; Carey C. J.; et al. SciPy 1.0: Fundamental Algorithms for Scientific Computing in Python. Nat. Methods 2020, 17, 261–272. 10.1038/s41592-019-0686-2. 32015543 PMC7056644

[ref56] Hunter J. D. Matplotlib: A 2D Graphics Environment. Comput. Sci. Eng. 2007, 9, 90–95. 10.1109/MCSE.2007.55.

