# Supplementary material for: Spatial Regulation of T-Cell Signaling by Programmed Death-Ligand 1 on Wireframe DNA Origami Flat Sheets
Source: ACS Nano. 2021 Feb 8;15(2):3441–52. doi: 10.1021/acsnano.0c10632 (PMC7905882; doi:10.1021/acsnano.0c10632)
Supplement: Supplementary file 1 — nn0c10632_si_001.pdf [file nn0c10632_si_001.pdf]

Supporting Information

Spatial Regulation of T-Cell Signaling by Programmed Death-Ligand 1 on Wireframe DNA Origami Flat Sheets

Trixy Fang[†], Jonatan Alvelid[‡], Joel Spratt[†], Elena Ambrosetti[†], Ilaria Testa[‡] and Ana I. Teixeira[†]*

[†]Department of Medical Biochemistry and Biophysics, Karolinska Institute, 17165, Stockholm, Sweden

[‡]Department of Applied Physics and Science for Life Laboratory, KTH Royal Institute of Technology, 100 44, Stockholm, Sweden

*Address correspondence to ana.teixeira@ki.se

Additional Methods

Reducing SDS-PAGE

Purified protein-oligo conjugates and unconjugated proteins (4 μM) were mixed with 1× NuPAGE Reducing Agent and 1× NuPAGE LDS sample buffer (ThermoFisher Scientific) and heated at 70°C for 10 min. The samples were run in 4-12% NuPAGE Bis-Tris gel together with SeeBlue Plus 2 Pre-stained Protein Standard in 1× NuPAGE MES SDS Running Buffer (ThermoFisher Scientific) at 200V for 35 min. Protein staining was performed using SimplyBlue Safe Stain (ThermoFisher Scientific) according to manufacturer's instructions.

Surface Plasmon Resonance (SPR) Assay

A Biacore T200 instrument (GE Healthcare) was used to perform SPR experiment. Anti-histidine antibodies (GE Healthcare) were immobilized on CM5 sensor chips (GE Healthcare) *via* amine coupling reaction, according to the manufacturer's instructions. Histidine-tagged PD-1 (R&D systems) was then passed over the surface and captured through the binding to the antibodies. HBS-EP+ (GE Healthcare) was used as running buffer. After the capture of PD-1, a stabilization time of 15 min was introduced to reach a stable baseline. PD-L1 flat sheets were injected at a 5 nM concentration in running buffer (HBS-EP+). Sensor surfaces functionalized with the same level of PD-1 were used for each PD-L1 flat sheet design. For every injection, a negative control (FS-empty) was performed.

Detection of Biotinylated Flat Sheets

Non-biotinylated and biotinylated flat sheets (20 nM) were incubated with 40 nM streptavidin-Alexa Fluor 647 conjugate (ThermoFisher Scientific) for 15 min at room temperature and detected by 2% agarose gel electrophoresis.

Retrovirus Production and Transduction

For STED imaging experiments, Jurkat E6-1 cells were retrovirally transduced to express SNAP-tagged PD-1. cDNA encoding human PD-1 (RDC0903, R&D Systems) was subcloned upstream of the SNAP-tag sequence in the pSNAP$_f$ vector (New England Biolabs) so that the resulting SNAP-tag would be located intracellularly. The PD-1-SNAP cDNA was then cloned into a retroviral pMSCV (with neomycin/G418 resistance) vector, which was a generous gift from Stephen L. Lessnick (Nationwide Children's Hospital). The recombinant virus vector was then sent to Vector Facility in Lund University for retrovirus production. To transduce the Jurkat T cells, 2×10^5 cells were infected at a multiplicity of infection (MOI) of 20 in complete RPMI medium containing 10 mM HEPES and 8 µg/ml polybrene (Sigma-Aldrich). The cells were incubated for 3 h at 37°C, 5% CO_2 before addition of fresh complete RPMI medium containing 10 mM HEPES to dilute the polybrene concentration to 4 µg/ml. The virus-cell mixture was incubated for 45 h at 37 °C, 5% CO_2 with a change of medium after 24 h. The transduced cells were then exchanged to selection medium (complete medium with 800 µg/ml G418 antibiotic) which was changed every 2 days for a period of 14 days.

Monoclonal PD-1-SNAP cell populations were isolated by the limiting dilution method in which single cells were plated in each well of a 96-well plate and checked for colony formation for 7-14 days.

Quantification of PD-1 by Flow Cytometry

The monoclonal PD-1-SNAP Jurkat cell population was tested for PD-1 expression with PE mouse anti-human PD-1 (560795, BD Biosciences) staining and analyzed by flow cytometry. The membrane expression of PD-1 was also quantified using BD Quantibrite beads (BD Biosciences) according to manufacturer's instructions. Samples were run on FACS Canto II flow cytometer and analyzed with FlowJo software (BD Biosciences).

PD-1 and SNAP Expression by Western Blot

To check for PD-1 and SNAP membrane expression, 2 × 10^6 PD-1-SNAP cells were lysed using Mem-PER Plus Membrane Protein Extraction kit (ThermoFisher Scientific). Membrane lysates were mixed with 1× NuPAGE LDS Sample Buffer (ThermoFisher Scientific), heated at 70 °C for 2 min and loaded into NuPAGE Novex 4-12% Bis-Tris gels with 10 μg lysate in each well. The gels were run at 200 V for 40 min in 1× NuPAGE MES SDS running buffer. Proteins were transferred onto nitrocellulose membranes of 0.2 μm pore size in transfer buffer (25 mM Tris, 190 mM glycine, 20% methanol (v/v) for 2 h, 30 V at 4°C. To visualize total protein content in cell membrane lysates, membranes were first stained with 1% (w/v) Ponceau S (Sigma-Aldrich) in 5% acetic acid for 5 min. Destaining was performed in 1×TBS with 0.1% Tween-20 (TBST) and membranes were blocked with 3% BSA in TBST. The following primary antibodies were used: anti-PD-1 mouse monoclonal (NAT105) (ab52587, Abcam), anti-SNAP-tag rabbit polyclonal antibody (P9310, NEB) and anti-sodium potassium ATPase antibody (EP1845Y) (ab76020, Abcam). Horseradish peroxidase-conjugated secondary antibodies used were: goat anti-mouse IgG (H+L) (#62-6520, ThermoFisher Scientific) and goat anti-rabbit IgG (H+L) (#32460, ThermoFisher Scientific). Proteins were detected using Pierce ECL Plus Western Blotting substrate (#321132, ThermoFisher Scientific) and imaged on ImageQuant LAS 4010 system (GE Healthcare).

Human IL-2 ELISA

PD-1-SNAP cells (2 × 10^5 cells per well in triplicates) were stimulated on a 96-well plate that was surface coated with 5 μg/ml anti-CD3ε IgG2a (OKT3, eBioscience) and 10 μg/ml human PD-L1 His-tag (9049-B7-100, R&D Systems). Soluble mouse anti-human CD28 /#555725, BD Biosciences) at 5 μg/ml were added after cell seeding. Cells were incubated at 37 °C, 5% CO_2 for 24 h. Supernatant from triplicates were pooled and human IL-2 was quantified using Human IL-2 DuoSet ELISA (DY202-05, R&D Systems).

Real-time Quantitative Reverse Transcription PCR (qRT-PCR)

PD-1-SNAP cells (3×10^4 cells in duplicates) were stimulated with FS-empty, FS-anti-CD3-CD28, FS-PD-L1-13 and FS-PD-L1-200 in a similar manner as PD-1-NFAT cells for the luciferase reporter assay. After incubation for 3 h, the cells were processed using the TaqMan Fast Advanced Cells-to-Ct kit and TaqMan Cells-to-Ct Control kit (ThermoFisher Scientific), following manufacturer's guidelines. TaqMan Gene Expression Assays used was IL-2 (Hs00174114_m1) and reactions were run on a StepOne Plus Real Time PCR system. Relative gene expression level (Ct of target gene normalized to beta-actin endogenous control gene and then Ct of FS-α-CD3-CD28) was calculated using the comparative Ct method formula $2^{-\Delta\Delta Ct}$.

Confocal Imaging and Analysis of Flat Sheet Colocalization

To detect the flat sheets with confocal microscopy, FS-α-CD3-CD28, FS-empty, FS-PD-L1-13 and FS-PD-L1-200 containing modified and protruding staples for fluorophore-oligo detection were produced (Table S5). Alexa Fluor 488-labeled oligos were added at 30-fold molar excess to hybridize complementarily to protruding ends of staples in FS-α-CD3-CD28. In the same manner, Alexa Fluor 594-labelled oligos were used to label FS-empty, FS-PD-L1-13 and FS-PD-L1-200. Excess fluorophore-oligos were removed together with unbound protein-oligo conjugates by Sepharose purification. The two-color confocal imaging of the flat sheets was performed on the custom-built STED set-up as described in the Methods section. Alexa Fluor 488 and Alexa Fluor 594-labelled oligos were excited with a 510 nm pulsed diode laser (LDH-D-C-510, PicoQuant) and 561 nm pulse diode laser (PDL561, Abberior Instruments) respectively. The detection was performed with a fiber-coupled APD (SPCM-AQRH-14-FC, PerkinElmer), and by detecting the Alexa488 signal through one bandpass filter (D535/40x, Chroma) and the Alexa594 signal through another bandpass filter (ET615/30m, Chroma). The colocalization analysis was performed in a custom-written script in Python by calculating the Pearson correlation coefficient between the two detection channels for each image, using the implementation in the scipy package.

Table S1

DNA oligos for protein conjugations

Tag 1	5’CTCTCCTTCTTCCCTTTCTTT/AzideN 3’
Tag 2	5’TTCGACAGCATGAACATCAGC/AzideN 3’
Tag 3	5’AATCCGTTAGTTCGTTGAA/AzideN 3’

Table S2

Fluorescently labeled complementary DNA oligos for conjugation check

Alexa Fluor 647-cTag 1	5’AAAGAAAGGGAAGAAGGAGAG/3AlexF647N/3’
Alexa Fluor 647-cTag 2	5’TTCAACGAACTAACGGATT/3AlexF647N/3’
Alexa Fluor 488-cTag 3	5’GCTGATGTTCATGCTGTCGAA/3AlexF488N/3’

Table S3

Biotinylated and modified staples

Plate	Well	Sequence
1	A8	5’ BiosG/TTCGTCAGATGAATATAAACCATCGCCCACGCA 3’
1	D8	5’ BiosG/TTGCAGCACCGTAATCAGTACAGTAGCACCATTACCATT 3’
2	B2	5’ BiosG/TTAAGGAATTGAGGAAGGTTACCTCAAATATCAAACCCT 3’
2	G8	5’ BiosG/TTGCGGATTGACCGTAATGGAAGGCGATTAAGTTGGGTA 3’
2	G7	5’ CGGCCAGTGCCAAGCTTTCGTGGGAACAAACG 3’
2	A4	5’ AGAGGACAGATGAACGGTAGATTTTCAGGTTTAA 3’

Table S4

Protruding staples for FS-α-CD3

Plate	Well	Sequence
1	E9	5′ AAAGAAAGGGAAGAAGGAGAGATAGCTATCTTAGCCGAACAAAGTTACCAGAAGGAAATAGCA 3′
1	G1	5′ AAAGAAAGGGAAGAAGGAGAGTAAGTCCTGAACAATGTAGAAACCAATCAATAATACAATAGA 3′

Protruding staples for FS-PD-L1

Plate	Well	Sequence
1	E9	5′ GCTGATGTTCATGCTGTCGAAATAGCTATCTTAGCCGAACAAAGTTACCAGAAGGAAATAGCA 3′
1	G1	5′ GCTGATGTTCATGCTGTCGAATAAGTCCTGAACAATGTAGAAACCAATCAATAATACAATAGA 3′

Protruding staples for FS-α-CD3-CD28

Plate	Well	Sequence
1	E7	5′ AAAGAAAGGGAAGAAGGAGAGTAATAACGGAATACGATTAAAGGGATTTTA 3′
1	E8	5′ AAAGAAAGGGAAGAAGGAGAGGCGCCTGTTTATCAGAAACCGAGGAAACGCAA 3′
1	E11	5′ TTCAACGAACTAACGGATTGCAAGAAACAATCGGCTGTCTTTCCTTATC 3′
1	F12	5′ TTCAACGAACTAACGGATTATTCCAAGAACGGGTATTATTTAATGGTTTGAAAT 3′

Protruding staples for FS-PD-L1-13

Plate	Well	Sequence
1	E7	5′ GCTGATGTTCATGCTGTCGAATAATAACGGAATACGATTAAAGGGATTTTA 3′
1	E8	5′ GCTGATGTTCATGCTGTCGAAGCGCCTGTTTATCAGAAACCGAGGAAACGCAA 3′
1	E11	5′ GCTGATGTTCATGCTGTCGAAGCAAGAAACAATCGGCTGTCTTTCCTTATC 3′
1	F12	5′ GCTGATGTTCATGCTGTCGAAATTCCAAGAACGGGTATTATTTAATGGTTTGAAAT 3′

Protruding staples for FS-PD-L1-40

Plate	Well	Sequence
1	F10	5′ GCTGATGTTCATGCTGTCGAACATTACCGCGCCCAATAGGAGACTACCTTTTTA 3′
1	F11	5′ GCTGATGTTCATGCTGTCGAAACCTCCGGCTTAGGTTGACCAAGTACCGCACTCATC 3′
2	C10	5′ GCTGATGTTCATGCTGTCGAATTTATAATCAGTGAGGCCCCTCGTTAGAATC 3′
2	C11	5′ GCTGATGTTCATGCTGTCGAAAGAGCGGGAGCTAAACAGGAGGCCCGCCGCGCTTAATGCGCCG 3′

Protruding staples for FS-PD-L1-200

Plate	Well	Sequence
1	A1	5′ GCTGATGTTCATGCTGTCGAAGACTAAAGACTTTTTCATGAGAGAAGTTTCCATTAAACGGGT 3′
2	H10	5′ GCTGATGTTCATGCTGTCGAAATCGATGAACGGTAATCGTAAATCAATATGATATTCAACC 3′
2	H11	5′ GCTGATGTTCATGCTGTCGAATTTTGAGAGATCTACAAAGGCTATCAGGTCATTGCCCAAGAGA 3′
2	H12	5′ TAAAAATTTTTAGAACCCTCAAAATGCCGGAGAGGGTAGCTAT 3′
3	B11	5′ GCTGATGTTCATGCTGTCGAAAAAATACGTAATGCCACTACGAAATAAATTGTGTCGAAATCCGCG 3′

Table S5

Fluorophore-modified oligos for confocal microscopy

5′GTTCGTAAGCCTCGCATGAGCCC/3AlexF488N/3′
5′GTTCGTAAGCCTCGCATGAGCCC/3AlexF594N/3′

Protruding and modified staples for fluorophore-oligo detection

Plate	Well	Sequence
1	B11	5′ GGGCTCATGCGAGGCTTACGAACTTTCCAGAGCCTAATTTGCCTCAGGAGGTTTAGTA 3′
1	C9	5′ GGGCTCATGCGAGGCTTACGAACAGCAAGGCCGGAAACGTCGGAAAGCGCAGTCTCT 3′
1	F5	5′ TTTTTTGTTTAACGTCAACCAACGCTAACGAGCGTC 3′
2	A9	5′ GGGCTCATGCGAGGCTTACGAACGAACAAAGAAACCACCAGAACGATAAAAACCAAAA 3′
2	B4	5′ GGGCTCATGCGAGGCTTACGAACCAATCAATATCTGGTCAGAACACCGCCTGCAACAGTG 3′
2	C5	5′ GGGCTCATGCGAGGCTTACGAACAGCTGTTTCCTGTGTGAAAAGCCATTGCAACAGGA 3′
2	D5	5′ GGGCTCATGCGAGGCTTACGAACTCTTTTCACCAGTGAGACAAGCCGGCGAACGTGG 3′
2	E7	5′ TTACACTGGTGTGTTCATTCGTAATCATGGTCAT 3′
2	H8	5′ GATTAAGACGCTGAGATAACATTATCATTTTGCG 3′

References

1. Rosenbluth, M.J.; Lam, W.A.; Fletcher, D.A. Force Microscopy of Nonadherent Cells: A Comparison of Leukemia Cell Deformability. Biophys. J. **2006**, 90, 2994–3003.

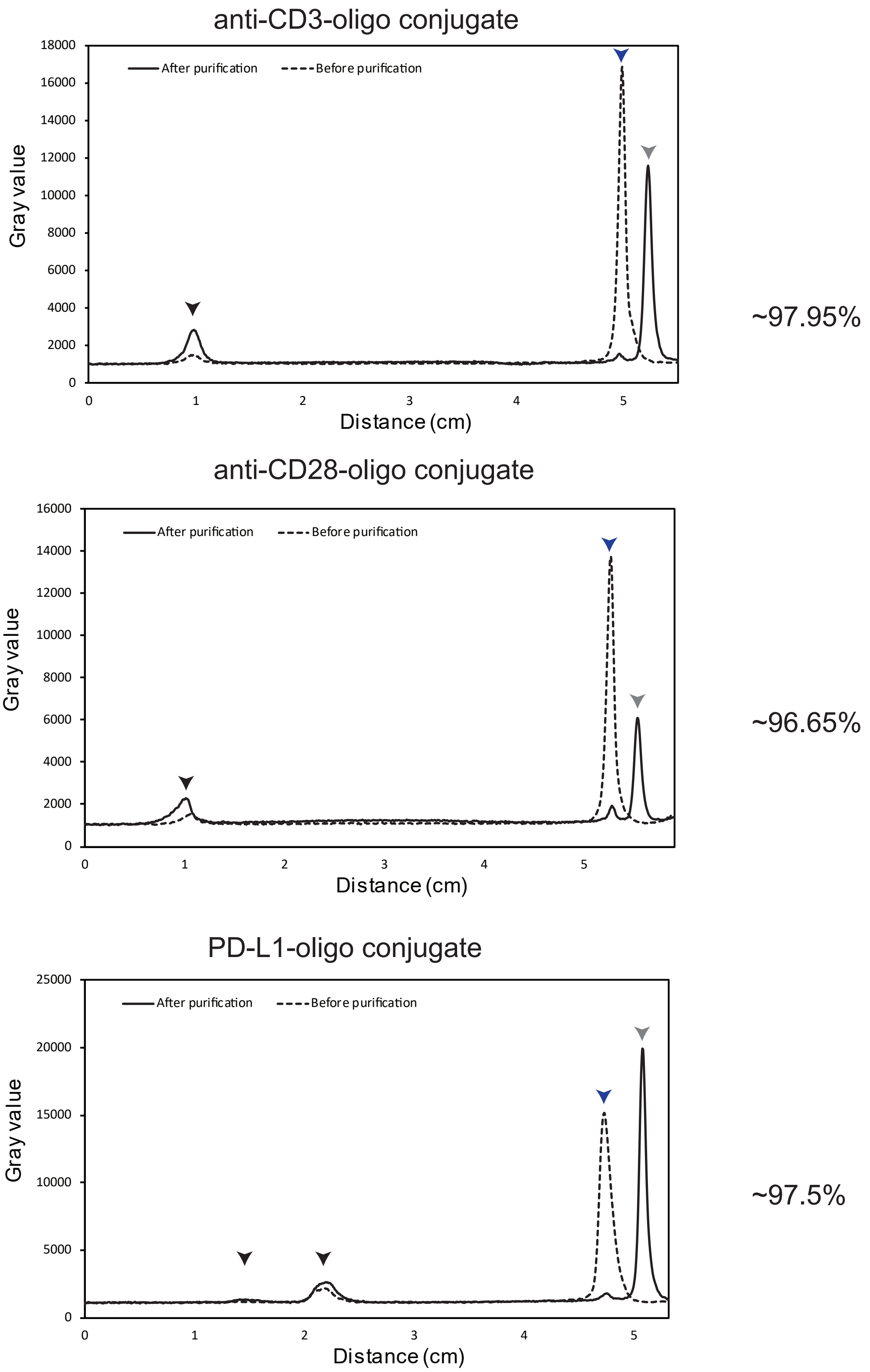

Figure S1. Line plots obtained from before and after purification lanes in fluorescence native PAGE images in Figure 1b (i)-(iii). Band peaks of protein-oligo conjugates (black arrowheads), azide-oligo/ fluorophore-oligo dimers (blue arrowheads) and flurophore-oligos (gray arrowheads) are shown. Estimated percentages of azide-oligos removed were calculated by taking the ratio of the area under the peaks of the azide-oligo/fluorophore-oligo dimer in both lanes.

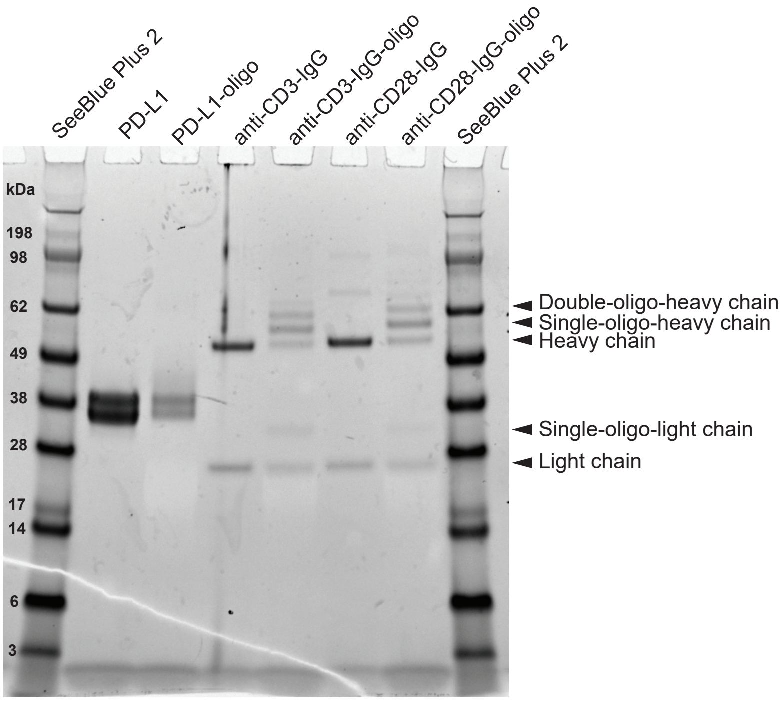

Figure S2. Reducing SDS-PAGE of protein-oligo conjugates. Black arrowheads indicate antibody fragments after reduction of disulfide bonds.

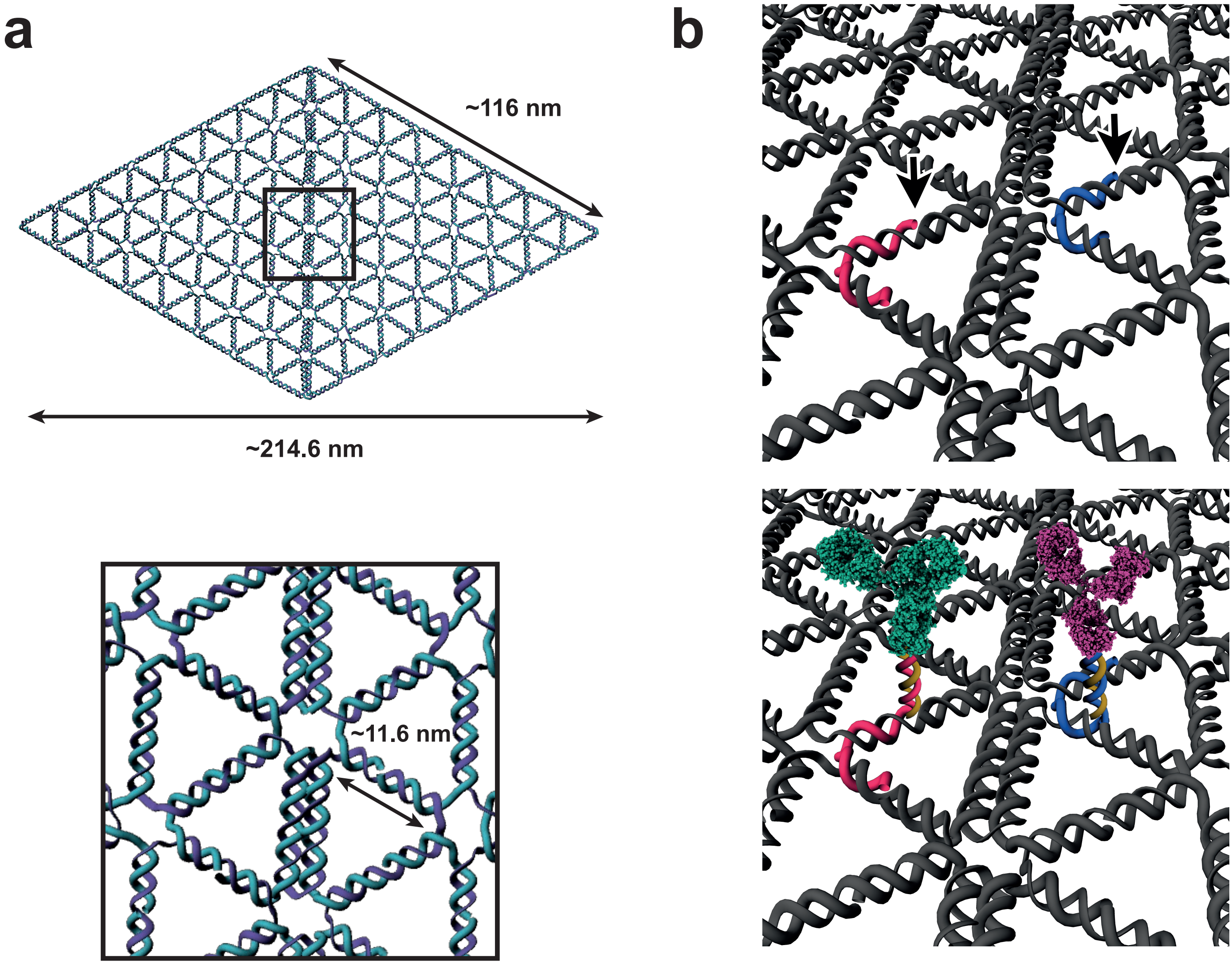

Figure S3. vHelix design of the DNA flat sheet nanostructure and principle of protein-flat sheet functionalization. **a** The p8064 scaffold strand (cyan) is folded by 216 staples (purple) based on 3-tessellation triangulated tiling. Black square shows a close-up image in the center. Theoretical dimensions of the flat sheet are calculated based on the distance of 0.34 nm between base pairs along the helix axis of B-form DNA. **b** To attach proteins onto the flat sheet, selected staples at known positions (pink and blue strands) were extended at the 5' end to hybridize to complementary oligos (yellow) conjugated to proteins.

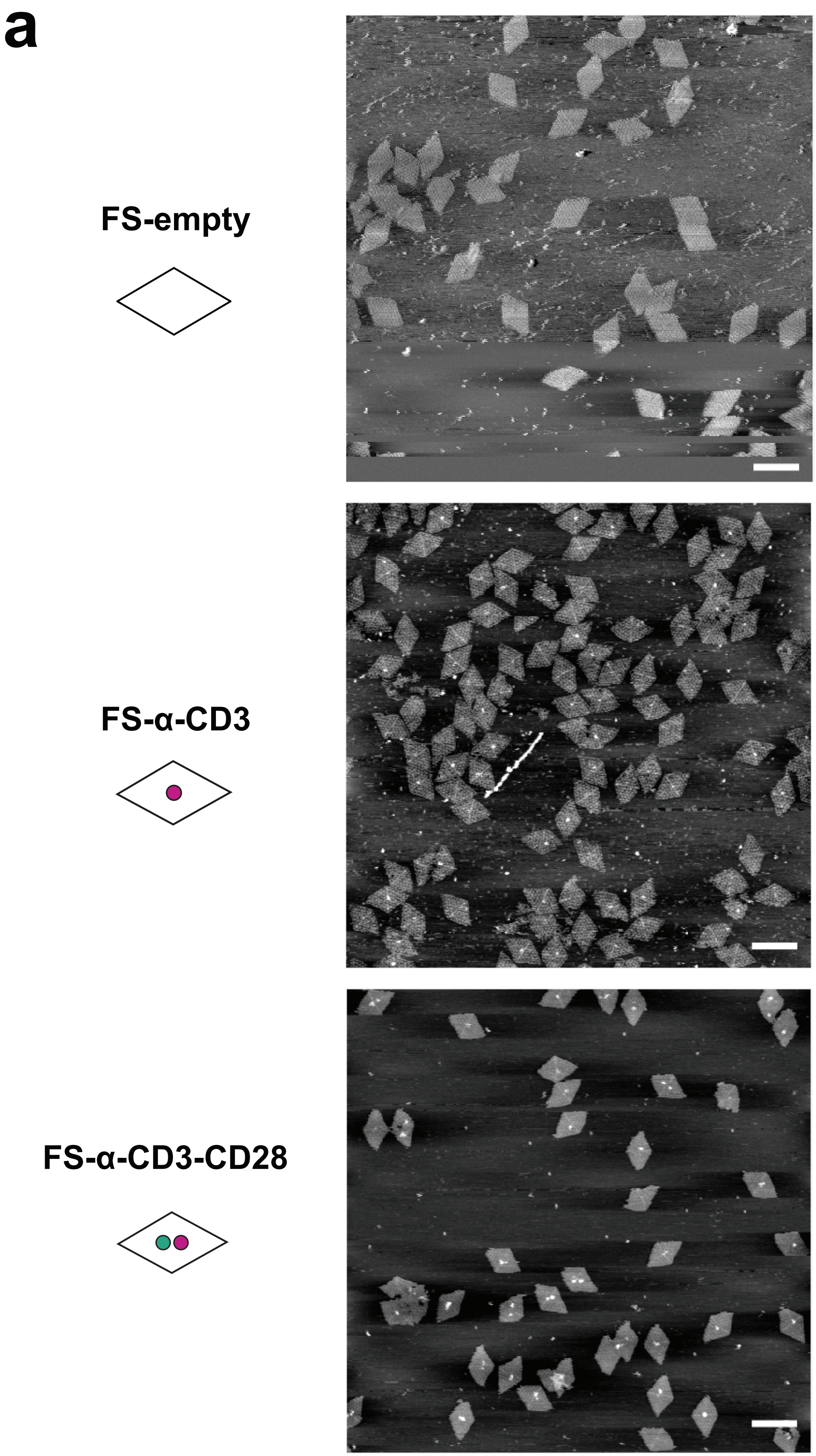
a
FS-empty
FS-α-CD3
FS-α-CD3-CD28

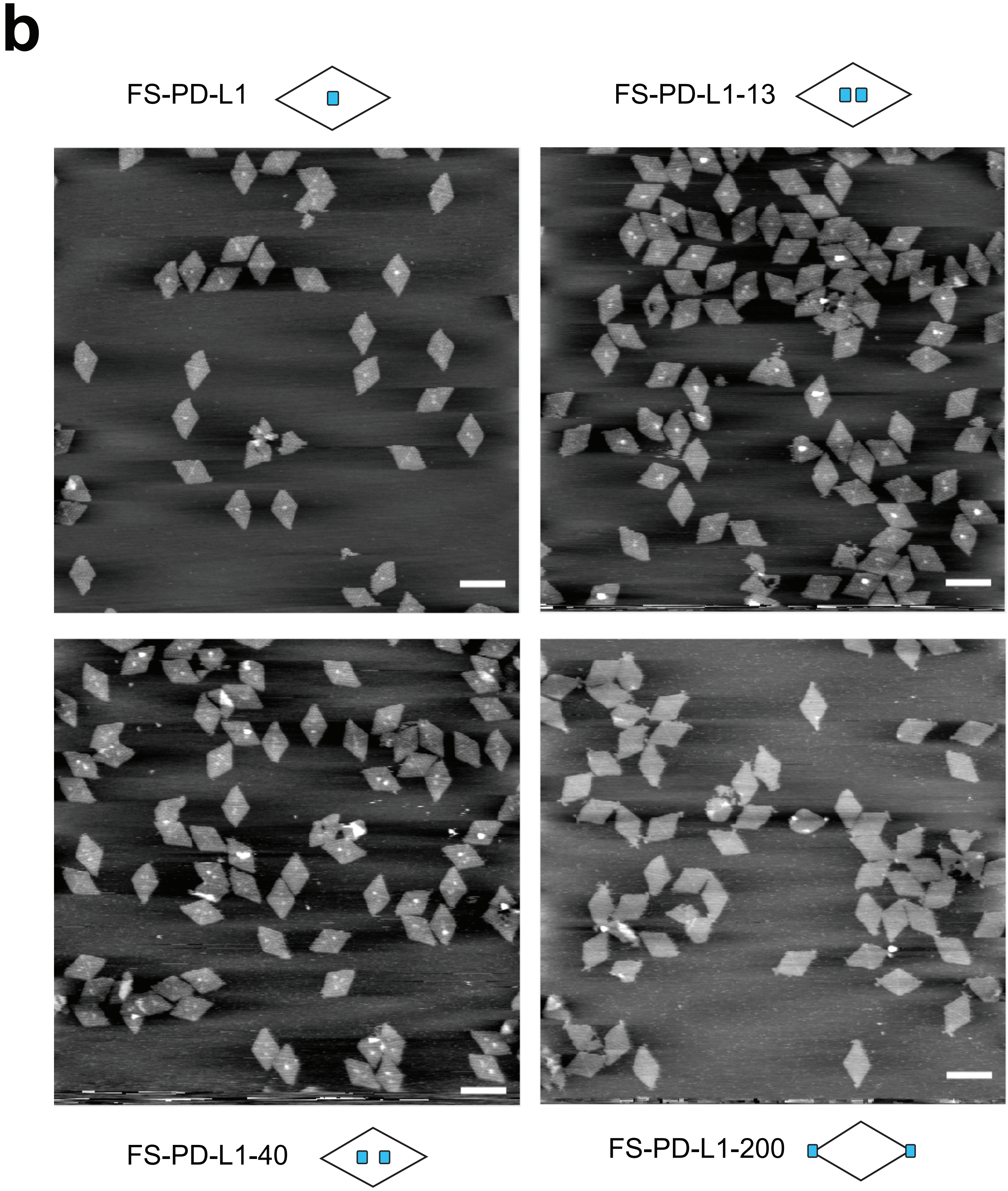

Figure S4. AFM imaging of protein-DNA flat sheets. Representative 2x2 µm images of **a** empty and antibody-flat sheets and **b** different PD-L1 flat sheet designs. Scale bar = 200 nm.

a

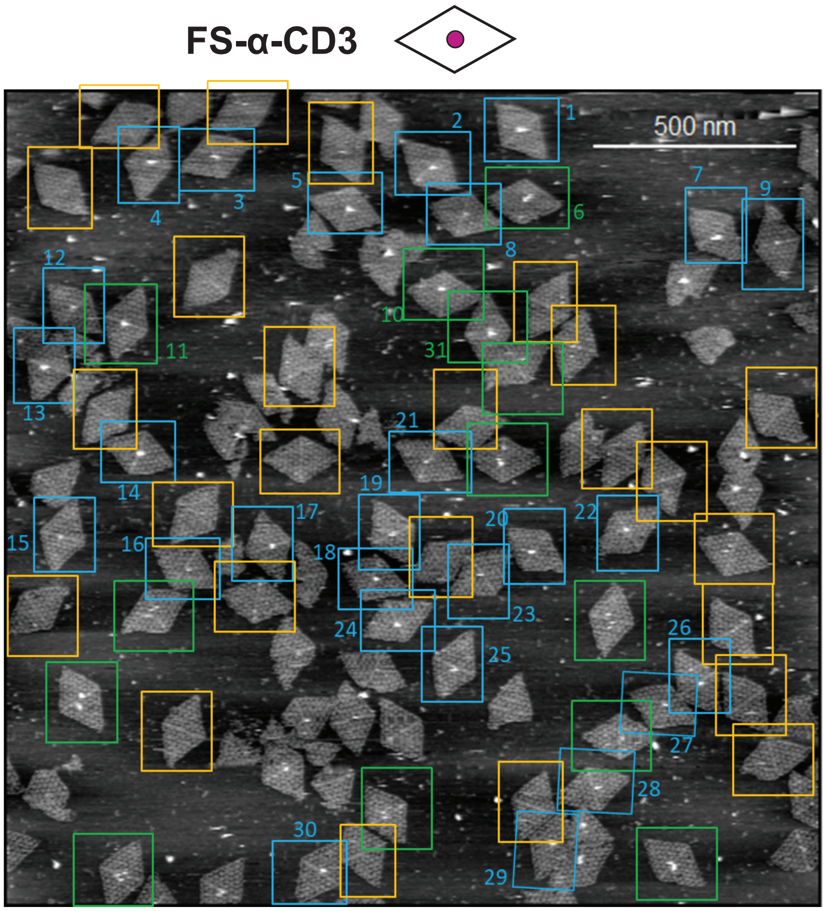

b

Flat sheet number	Peak width (nm)	Number of proteins
1	29.7	1
2	23.8	1
3	28	1
4	25.9	1
5	30.8	1
6	34.5	Undetermined
7	30.6	1
8	27.1	1
9	16.1	1
10	34	Undetermined
11	34	Undetermined
12	30.1	1
13	31.4	1
14	31.8	1
15	23.8	1
16	25.9	1
17	22.1	1
18	31.2	1
19	27.2	1
20	24.2	1
21	26.8	1
22	30.1	1
23	19.5	1
24	31	1
25	27.6	1
26	23.8	1
27	28	1
28	31.4	1
29	28	1
30	26.8	1
31	34.8	Undetermined

c

**Flat Sheet Occupancy**		
Number of Proteins	Count	%
0	**73**	**44.51**
1	**69**	**42.07**
Undetermined	**22**	**13.41**
	164	100

Figure S5. Estimation of protein occupancy for FS-α-CD3 design. **a** A 2 x 2 µm AFM image showing flat sheets that are quantified for protein occupancy by the observed blobs on each flat sheet (boxes). Fragmented, damaged or overlapping flat sheets are not counted. Scale bar = 500 nm. Flat sheets which showed more blobs than designed, large (> 32 nm) or nicked blobs are labeled with a green box. Flat sheets that have 1 blob are labeled with a blue box. Yellow boxes indicate empty flat sheets. Numbered boxes are analyzed by line trace profiling to obtain the peak (blob) widths. **b** Table showing the flat sheets in numbered boxes in **a** with the measured peak widths from their line trace profiles and estimated protein occupancy. Flat sheets showing peak widths of >32 nm or contain more blobs than designed were assigned to be “undetermined” in protein occupancy as the FS-α-CD3 design contains a single binding site. **c** Table showing the estimated protein occupancy (counts and percentages) of 164 flat sheets from three AFM images.

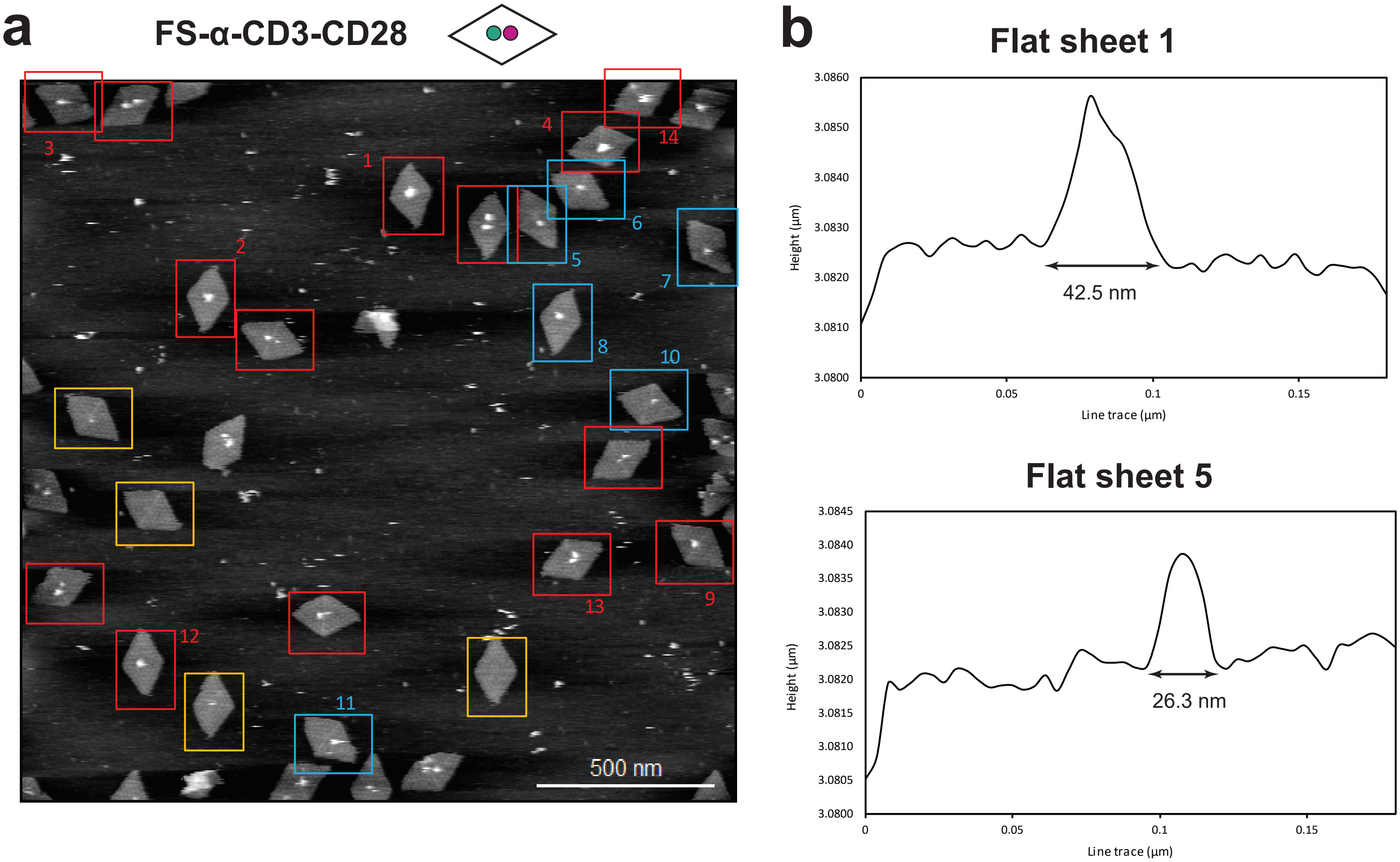

c

Flat sheet number	Peak width (nm)	Number of proteins
1	42.5	2
2	32.7	2
3	38.6	2
4	48.4	2
5	26.3	1
6	28.9	1
7	20.4	1
8	27.2	1
9	38.6	2
10	25.1	1
11	30.6	1
12	36.1	2
13	47.6	2
14	39.9	2

d

Flat Sheet Occupancy		
Number of Proteins	Count	%
0	**8**	**10.13**
1	**26**	**32.91**
2	**45**	**56.96**
	79	100

Figure S6. Estimation of protein occupancy for FS-α-CD3-CD28 design. **a** A 2 x 2 µm AFM image showing flat sheets that are quantified for protein occupancy by the observed blobs on each flat sheet (boxes). Fragmented, damaged or overlapping flat sheets are not counted. Flat sheets with red boxes indicate 2 blobs or large blobs (>32 nm), blue boxes indicate 1 blob and yellow boxes indicate empty structures. Flat sheets labeled with numbered boxes are analyzed by line trace profiling to obtain the peak (blob) widths. Scale bar = 500 nm. **b** Examples of line trace profiles of numbered flat sheets in **a** and peak widths (nm) as indicated. **c** Table showing the numbered flat sheets in **a** with measured peak widths from their line trace profiles and estimated protein occupancy. Flat sheets showing peak widths of >32 nm were assigned to be occupied wth 2 proteins. **d** Table showing the estimated protein occupancy (counts and percentages) of 79 flat sheets from three AFM images.

a

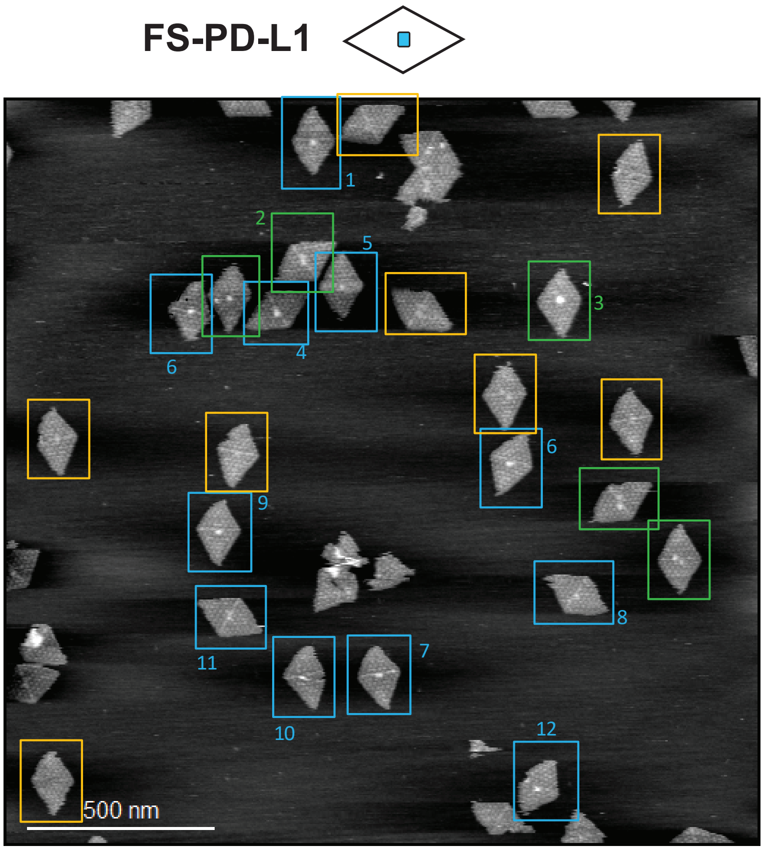

b

Flat sheet number	Peak width (nm)	Number of proteins
1	27.6	1
2	33.1	Undetermined
3	36.1	Undetermined
4	27.4	1
5	24.6	1
6	26.1	1
7	22.9	1
8	24.2	1
9	23.3	1
10	22.9	1
11	29.3	1
12	18.7	1

c

Flat Sheet Occupancy		
Number of Proteins	Count	%
0	**16**	**22.9**
1	**42**	**60.0**
2	**12**	**17.1**
	70	100

Figure S7. Estimation of protein occupancy for FS-PD-L1 design. **a** A 2 x 2 μm AFM image showing flat sheets that are quantified for protein occupancy by the observed blobs on each flat sheet (boxes). Fragmented, damaged or overlapping flat sheets are not counted. Scale bar = 500 nm. Flat sheets which showed more blobs than designed, large (> 32 nm) or nicked blobs labeled with a green box. Flat sheets in blue boxes indicate 1 protein and yellow boxes indicate empty structures. **b** Table showing the flat sheets in numbered boxes in **a** with the measured peak widths from their line trace profiles and estimated protein occupancy. Flat sheets showing peak widths of >32 nm or contain more blobs than designed were assigned to be “undetermined” in protein occupancy as the FS-PD-L1 design contains a single binding site. **c** Table showing the estimated protein occupancy (counts and percentages) of 70 flat sheets from three AFM images.

a

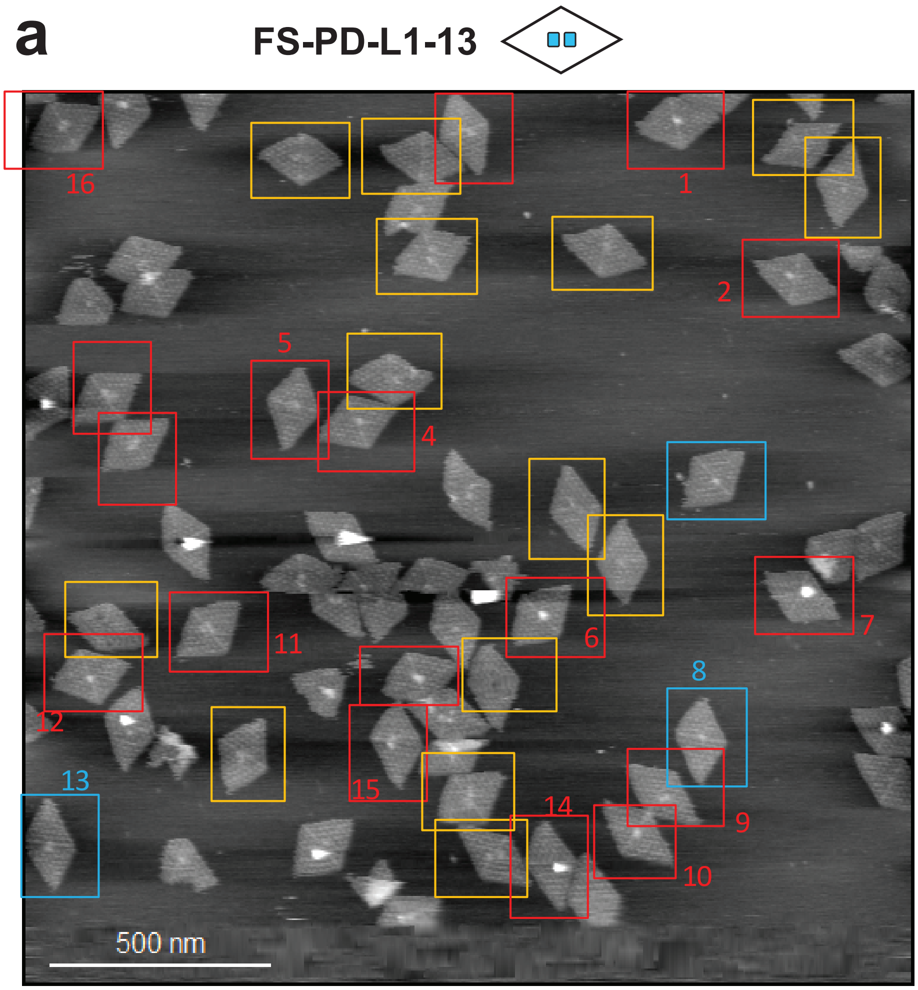

b

Flat sheet number	Peak width (nm)	Number of proteins
1	39.5	2
2	34	2
3	26.3	1
4	33.5	2
5	33.5	2
6	41.6	2
7	45.9	2
8	26.3	1
9	38.2	2
10	41.9	2
11	35.2	2
12	35.2	2
13	31.5	1
14	36.1	2
15	39.1	2
16	36.9	2

c

Flat Sheet Occupancy		
Number of Proteins	Count	%
0	**44**	**31.65**
1	**28**	**20.14**
2	**67**	**48.20**
	139	100

Figure S8. Estimation of protein occupancy for FS-PD-L1-13 design. **a** A 2 x 2 μm AFM image showing flat sheets that are quantified for protein occupancy by the observed blobs on each flat sheet (boxes). Fragmented, damaged or overlapping flat sheets are not counted. Scale bar = 500 nm. Flat sheets with red boxes indicate 2 blobs or large blobs (>32 nm), blue boxes indicate 1 blob and yellow boxes indicate empty structures. **b** Table showing the flat sheets in numbered boxes in **a** with the measured peak widths from their line trace profiles and estimated protein occupancy. Flat sheets showing peak widths of >32 nm were assigned to be occupied with 2 proteins. **c** Table showing the estimated protein occupancy (counts and percentages) of 139 flat sheets from three AFM images.

a

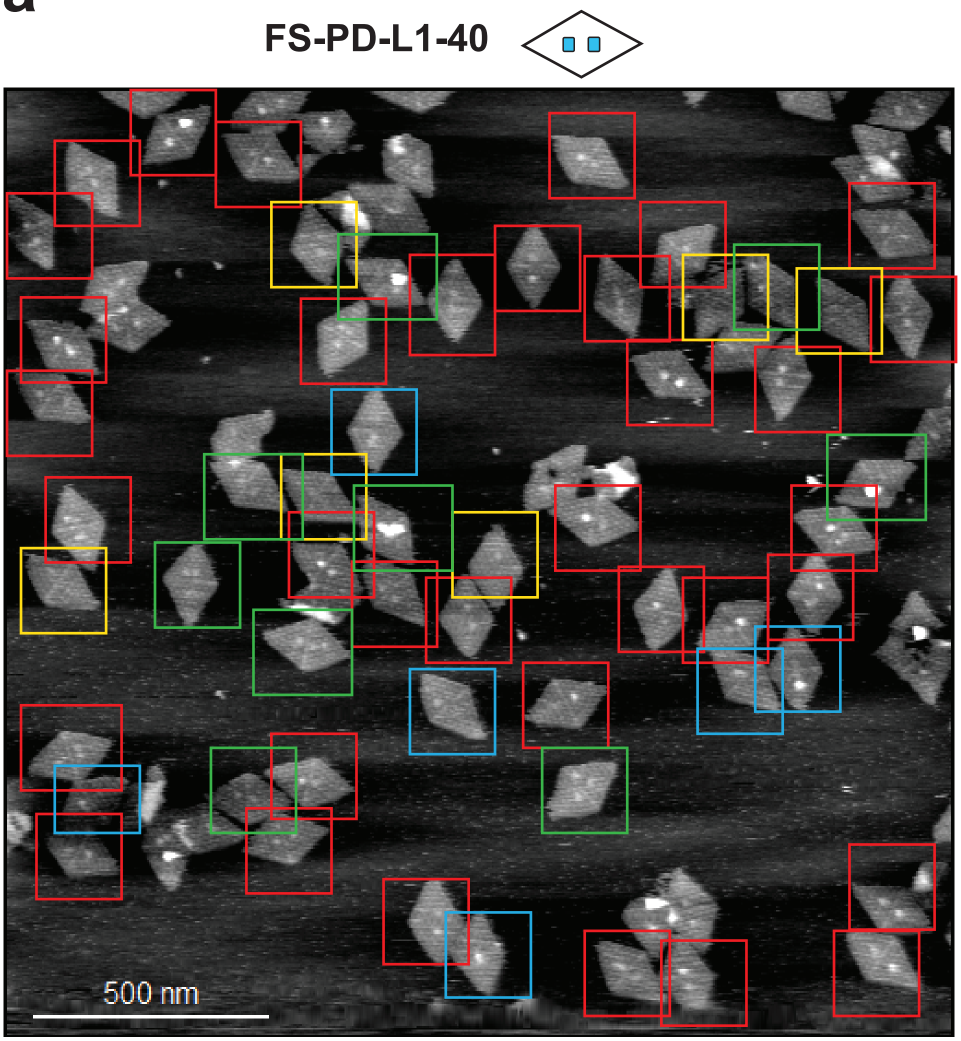

b

Number of Proteins	Counts
0	6
1	6
2	35
Undetermined	9
Total	56

c

Flat Sheet Occupancy		
Number of Proteins	Count	%
0	**14**	**10.22**
1	**14**	**10.22**
2	**89**	**64.96**
Undetermined	**20**	**14.60**
	137	100

Figure S9. Estimation of protein occupancy for FS-PD-L1-40 design. **a** A 2 x 2 µm AFM image showing flat sheets that are quantified for protein occupancy by the observed blobs on each flat sheet (boxes). Fragmented, damaged or overlapping flat sheets are not counted. Scale bar = 500 nm. Flat sheets which showed more blobs than designed, large (> 32 nm) or nicked blobs are labeled with a green box. Flat sheets that show 1 blob or 2 blobs are labeled with blue and red boxes respectively. Yellow boxes indicate empty structures. **b** Table showing the flat sheet counts in **a** with the observed protein occupancy or "undetermined" (green boxes). **c** Table showing the estimated protein occupancy (counts and percentages) of 137 flat sheets from three AFM images.

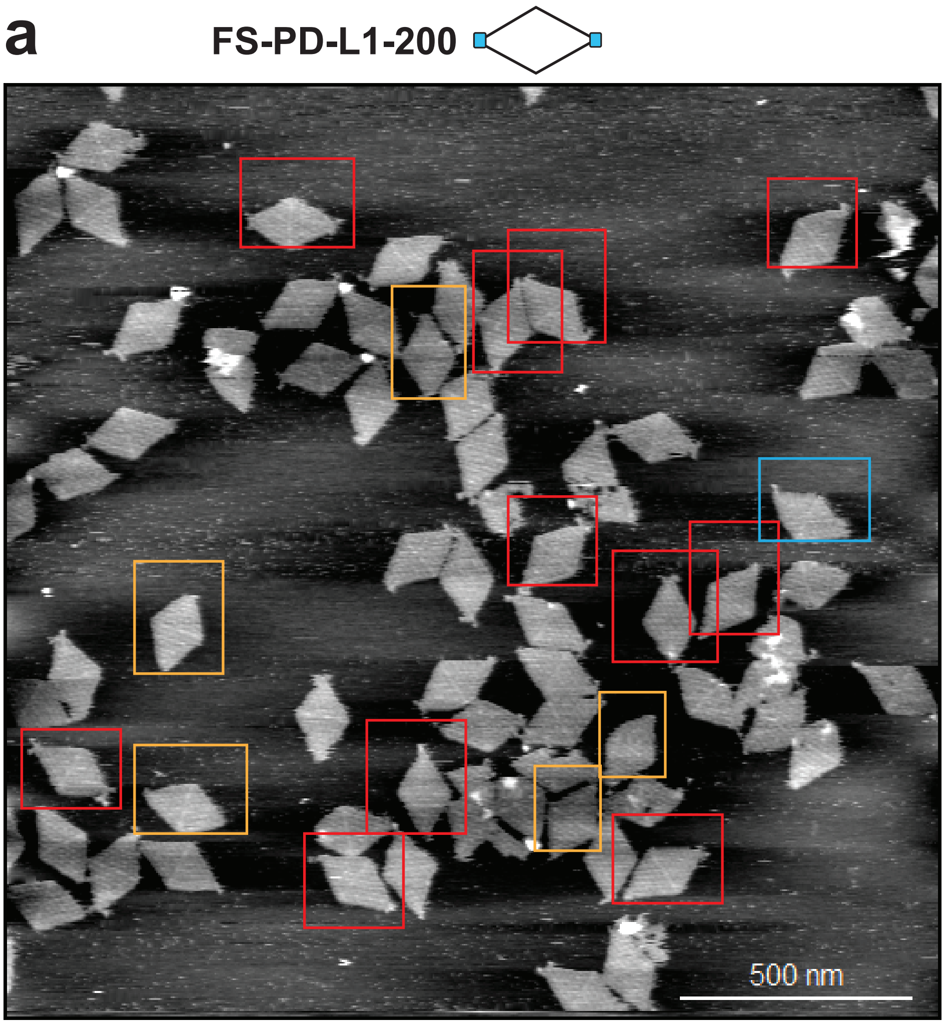

b

Number of Proteins	Counts
0	5
1	1
2	11
Total	17

c

Flat Sheet Occupancy		
Number of Proteins	Count	%
0	**13**	**25.0**
1	**6**	**11.5**
2	**33**	**63.5**
	52	100

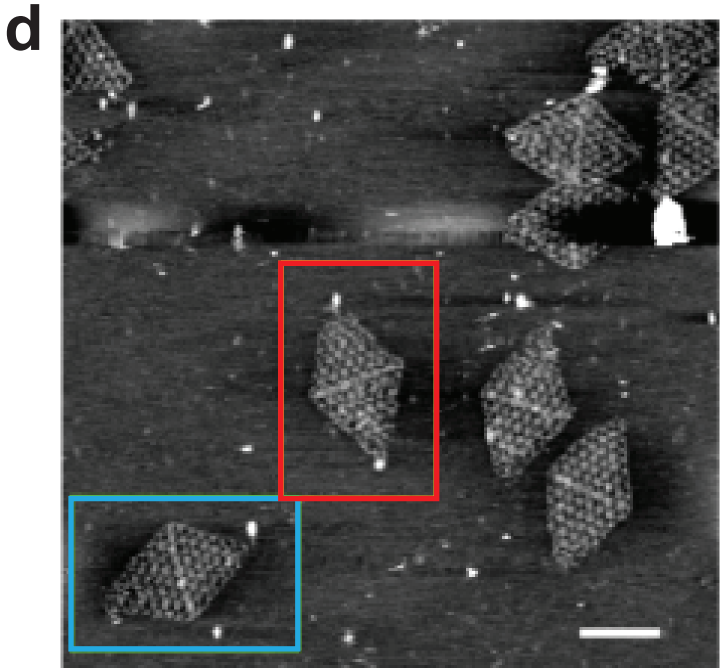

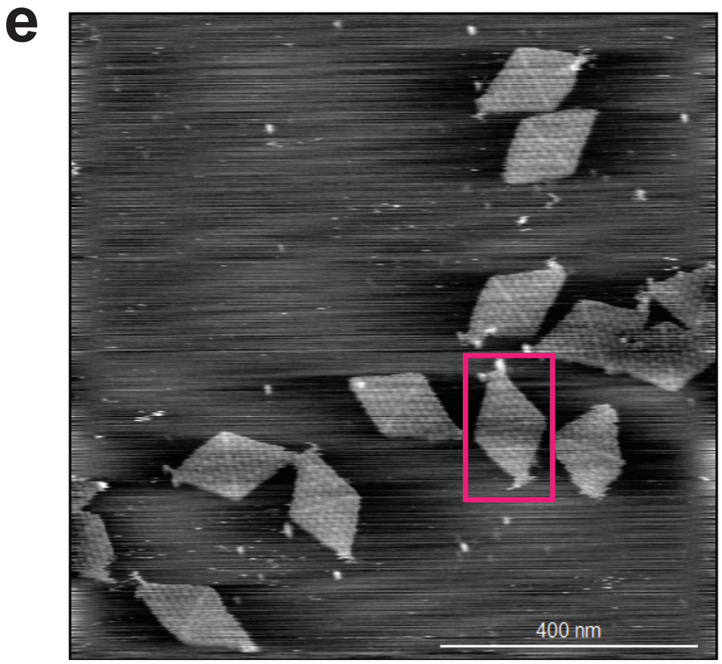

Figure S10. Estimation of protein occupancy for FS-PD-L1-200 design. **a** A 2 x 2 µm AFM image showing flat sheets that are quantified for protein occupancy by the observed blobs on each flat sheet (boxes). Scale bar = 500 nm. Flat sheets with red boxes indicate 2 blobs as designed, blue boxes indicate 1 blob and yellow boxes indicate empty flat sheets. **b** Table showing the flat sheet counts with the protein occupancy in **a**. **c** Table showing the estimated protein occupancy (counts and percentages) of 52 flat sheets from three AFM images. **d** AFM image (1 x 1 µm) showing a flat sheet with two blobs at the ends as designed (red box) and a flat sheet with one blob and one nicked end (blue box). Scale bar = 100 nm. **e** This design tend to show nicked/fragmented blobs at the ends of the flat sheet (pink box) with AFM imaging. Scale bar = 400 nm.

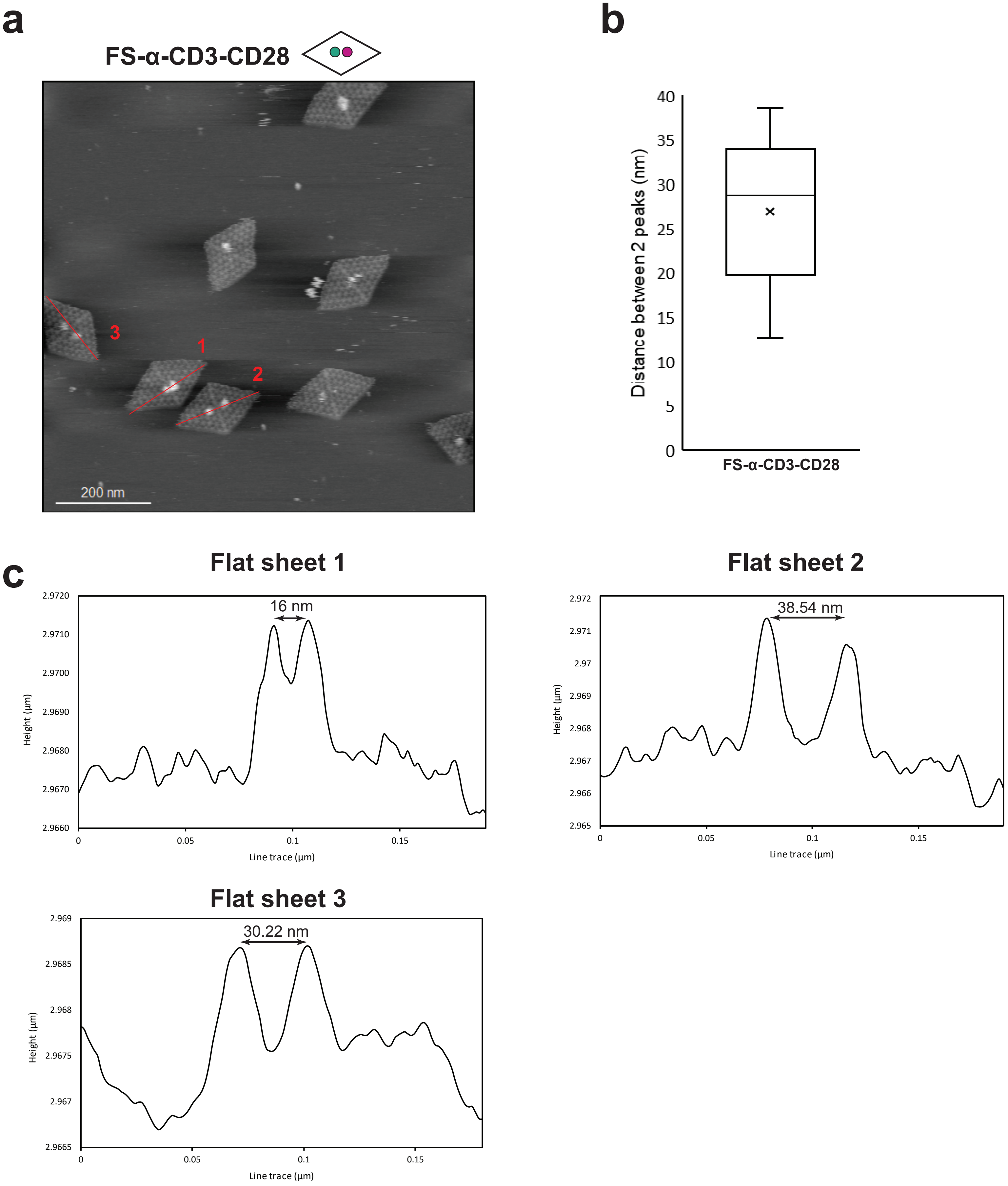

Figure S11. Distances between proteins for FS-α-CD3-CD28 design. **a** A 0.9 x 0.9 µm AFM image of FS-α-CD3-CD28 with numbered line traces (red) on flat sheets with 2 blobs. Scale bar = 200 nm. **b** A boxplot showing the distribution of distances between 2 peaks (blobs) of 25 flat sheets. Mean distance is indicated by the cross (26.82 nm). Whiskers shows the minimum and maximum, line in box shows the median. **c** Line trace profiles from image in **a** with distances measured by the maxima of two peaks.

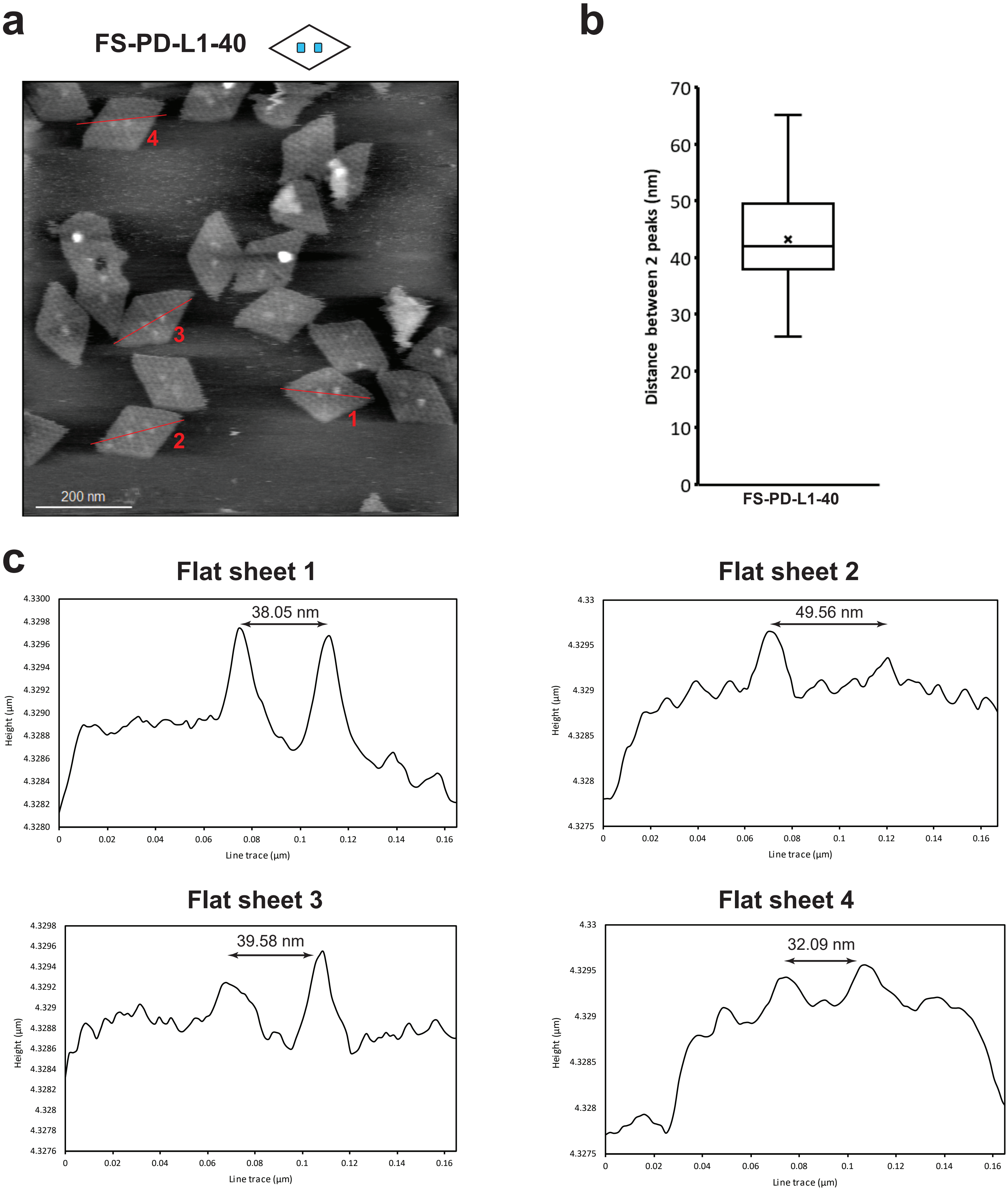

Figure S12. Estimation of distances between proteins for FS-PD-L1-40 design. **a** A 0.9 x 0.9 µm AFM image of FS-PD-L1-40 with numbered line traces (red) on flat sheets. Scale bar = 200 nm. **b** A boxplot showing the distribution of distances between 2 peaks (blobs) of 31 flat sheets. The mean distance is indicated by the cross (43.35 nm). Whiskers shows the minimum and maximum, line in box shows the median. **c** Line trace profiles from image in **a** with distances measured by the maxima of two peaks.

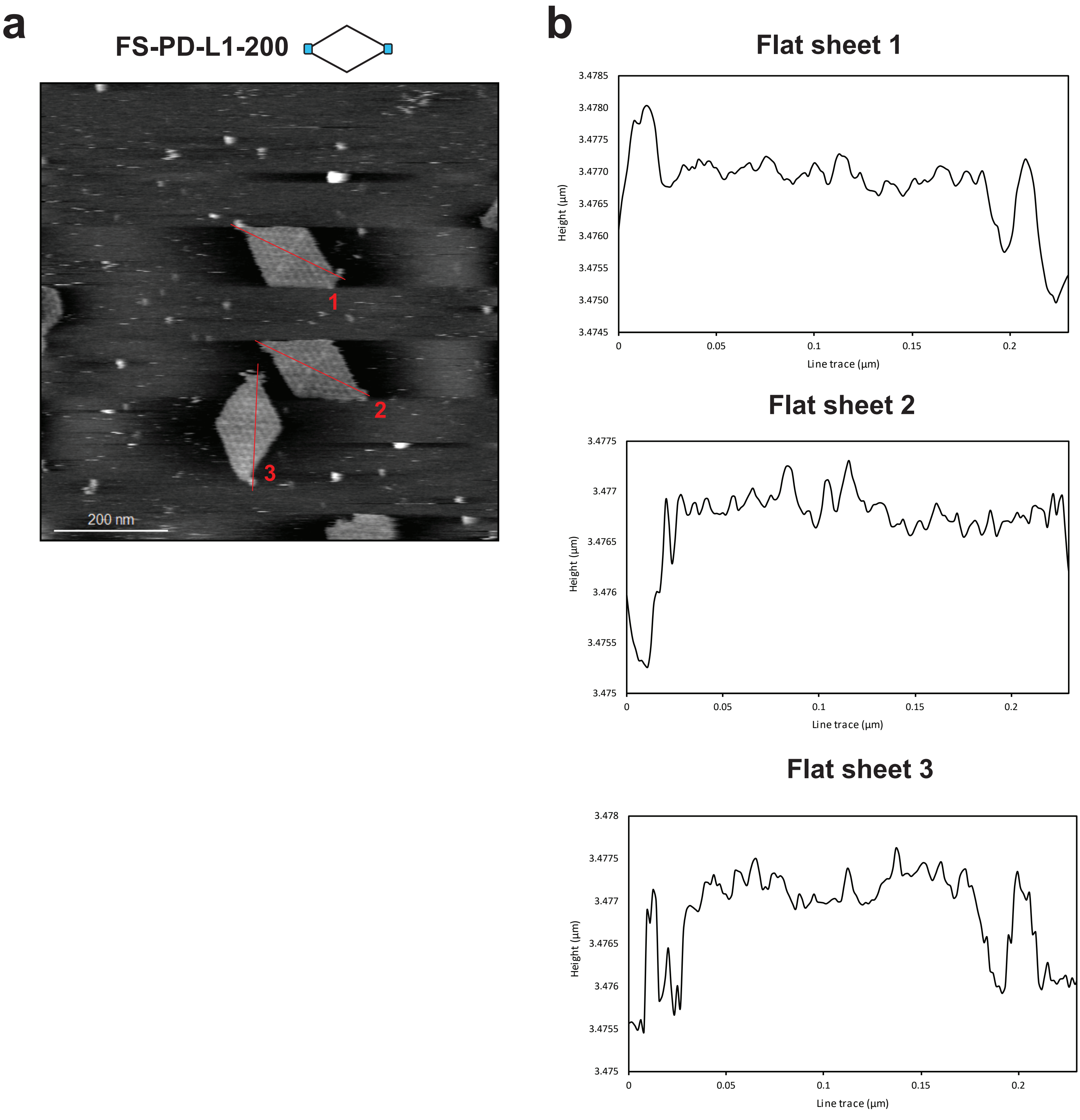

Figure S13. Estimation of distances between proteins for FS-PD-L1-200 design. **a** A 0.9 x 0.9 µm AFM image of FS-PD-L1-200 with numbered line traces (red) on flat sheets. Scale bar = 200 nm. **b** Line trace profiles of flat sheets from image in **a**. Distance between proteins could not be determined for this design as PD-L1 at the ends of the flat sheet tend to contact the mica surface, resulting in similar heights as the flat sheet plane.

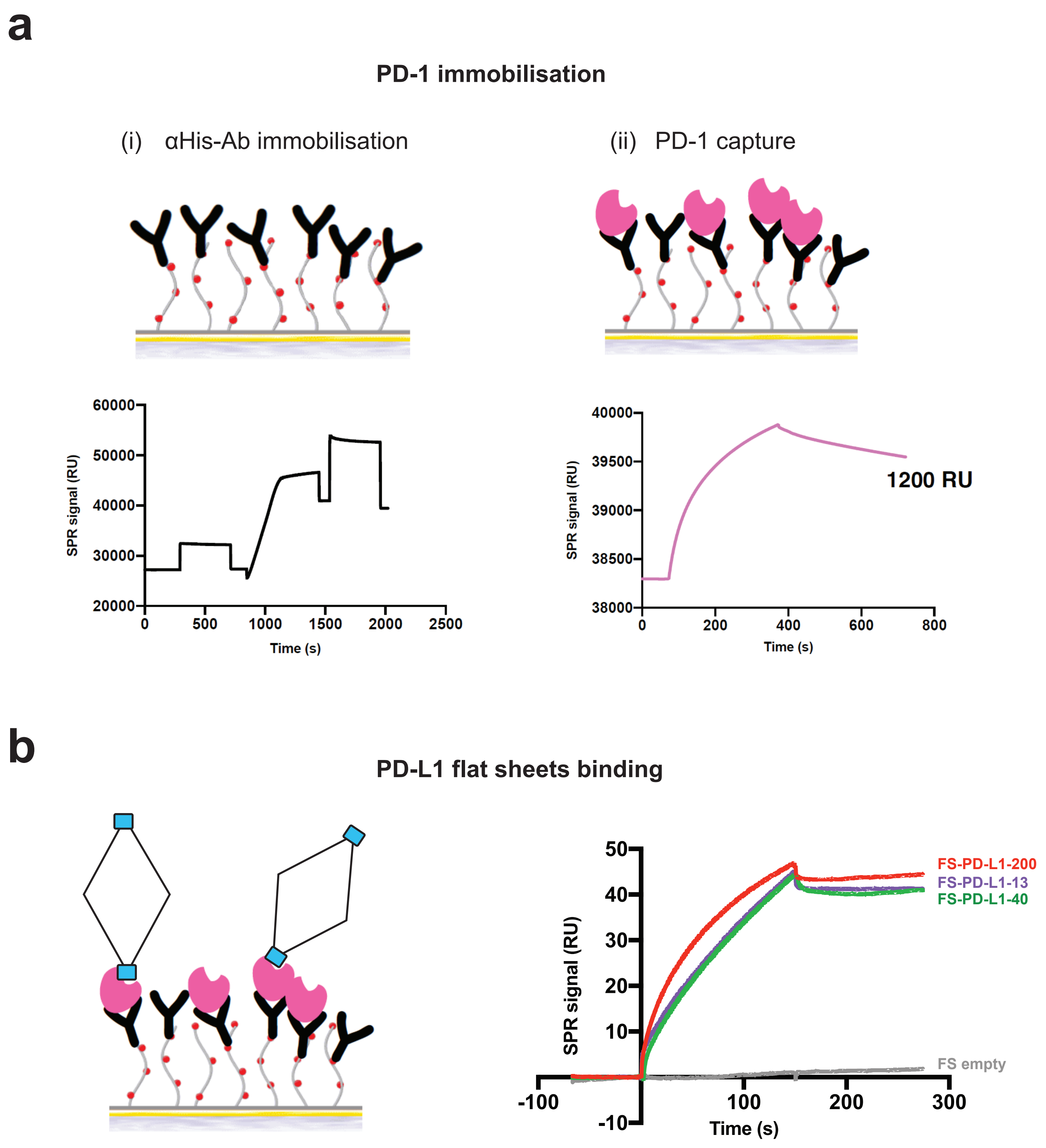

Figure S14. SPR validation of binding of PD-L1 flat sheets to PD-1. **a** PD-1 was immobilised on a SPR sensor surface by a capture approach. First, an anti-histidine antibody was covalently attached to the sensor surface (i) and histidine-tagged PD-1 was then captured (ii). **b** The different PD-L1 flat sheets (FS-PD-L1-13, FS-PD-L1-40, FS-PD-L1-200) and FS-empty as negative control were injected over the surface to verify the binding to PD-1.

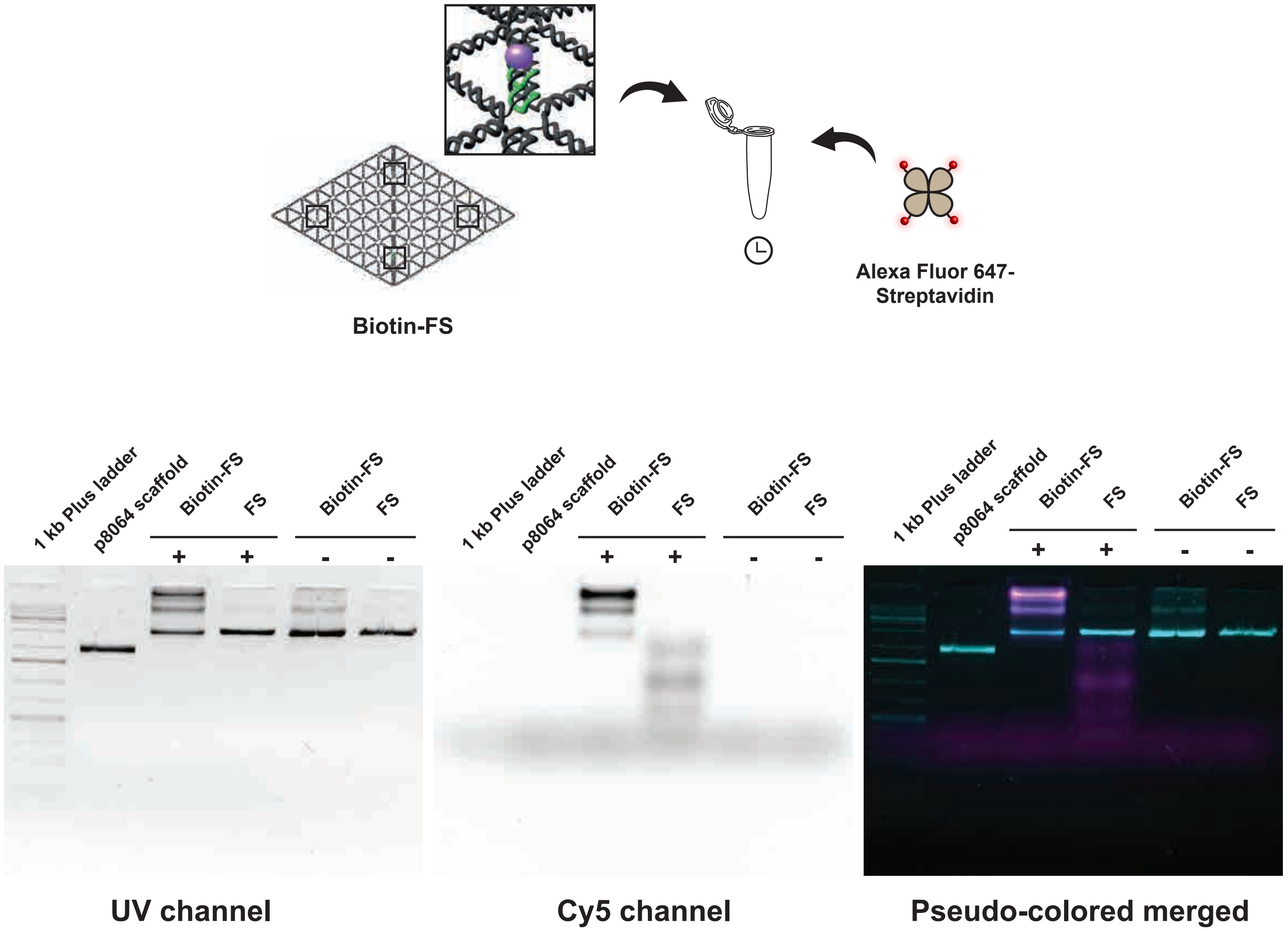

Figure S15. Detection of biotinylated flat sheets with fluorescently labeled streptavidin. Biotin-FS (20 nM) were mixed with 200 nM Alexa Fluor 647-streptavidin for 15 min at room temperature. Non-biotinylated FS and biotin-FS mixed with (+) and without (-) Alexa Fluor 647-streptavidin on a 2% agarose gel imaged by UV (left), Cy5 (middle) transillumination and then pseudo-colored with cyan (UV) and magenta (Cy5) (right).

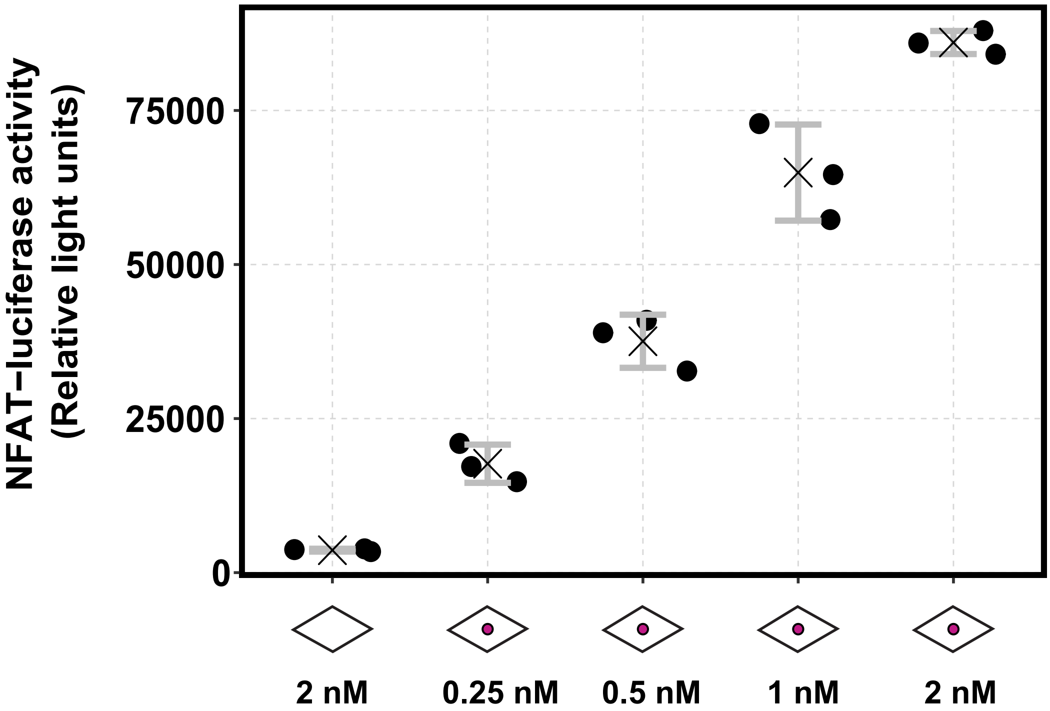

Figure S16. NFAT-luciferase activity of PD-1-NFAT cells stimulated with a series of FS-α-CD3 concentrations. Plot showing luciferase activity (relative light units) with increasing FS-α-CD3 concentrations. FS-empty of 2 nM concentration was used as a control. Each dot represents a technical replicate of 3000 cells. Mean and standard deviation are represented as crosses and error bars.

a

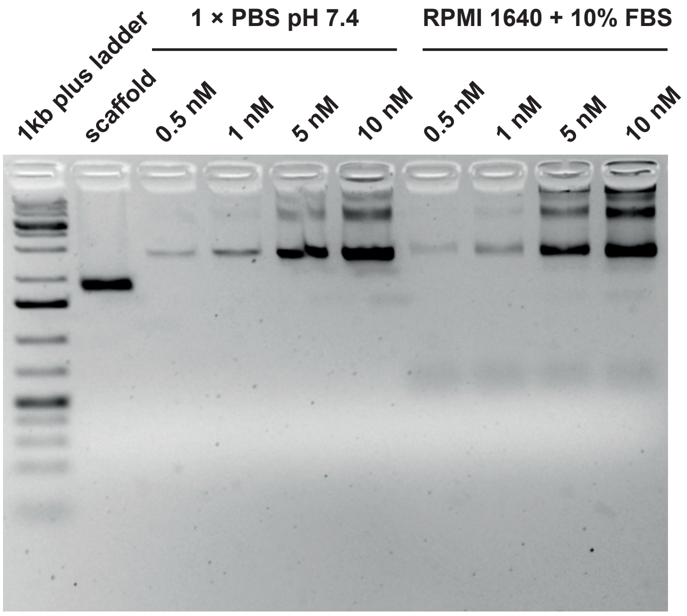

b

Flat sheet concentration (nM)	% yield
0.5	79.8
1	76.7
5	66.1
10	60.2

Figure S17. Integrity of flat sheets in 1×PBS and complete RPMI media.**a** 2% agarose gel electrophoresis of a concentration series of empty flat sheets in PBS or RPMI with 10% FBS after 3 hours at 37°C. **b** Yield estimation of flat sheets in complete RPMI media by calculating the intensity of gel bands in media as a fraction of the band intensities in PBS. ImageJ was used to measure the band intensites.

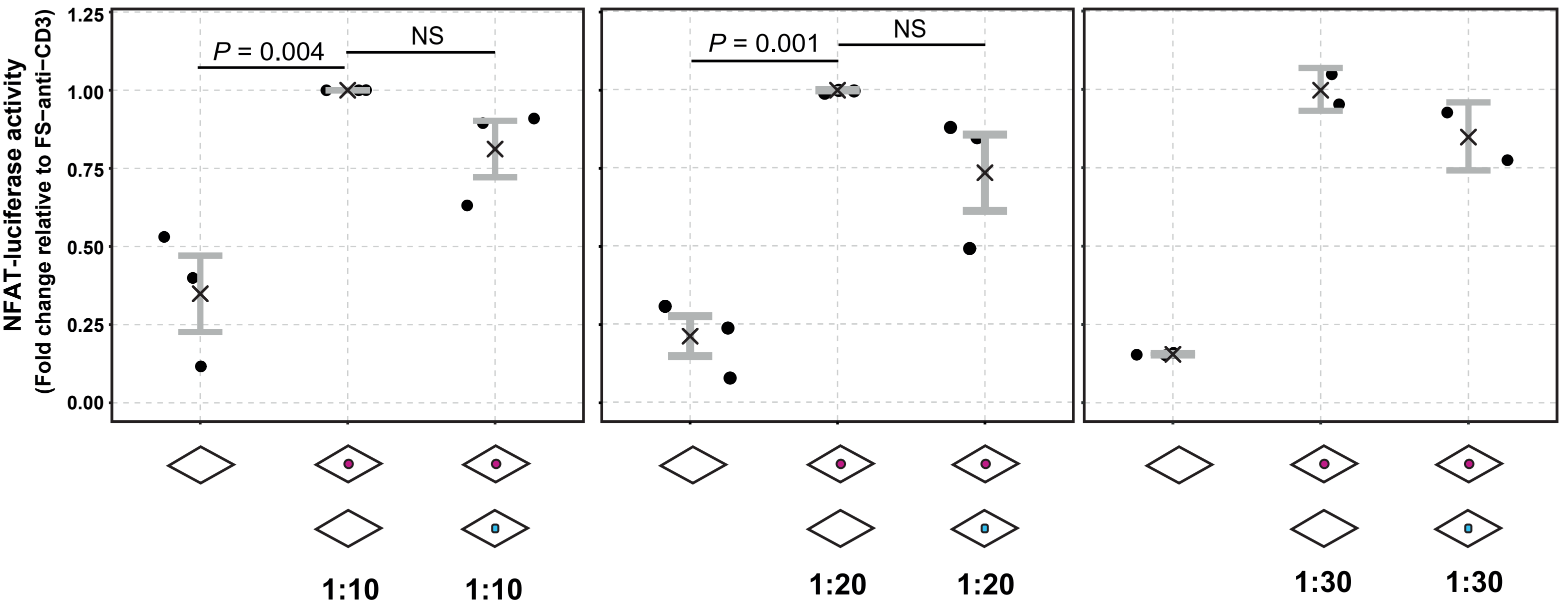

Figure S18. NFAT-luciferase activity of PD-1-NFAT cells stimulated with a series of FS-α-CD3 to FS-PD-L1 molar ratios. Fold changes were relative to cells stimulated with FS-α-CD3. For left and middle panels, data are 3 independent experiments (black dots, N = 3), with each dot representing the average reading of 3 technical replicates consisting of 3000 cells each. The mean and standard error of the mean are represented as crosses and error bars.
NS: Not significant. For the right panel, data is one experiment (N = 1) of 2 technical replicates (black dots) consisting of 3000 cells each. Mean and standard deviation are indicated as crosses and error bars.

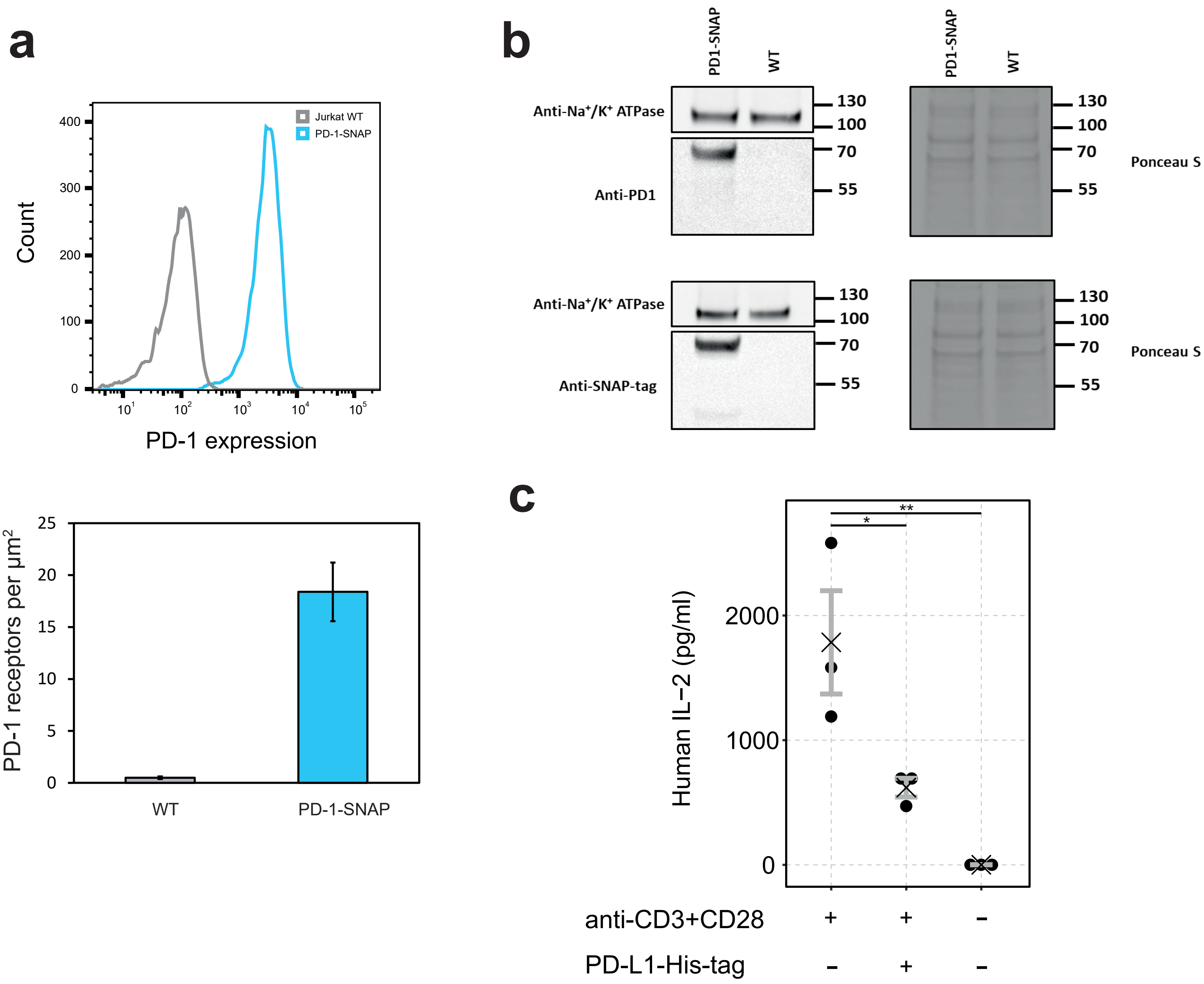

Figure S19 . PD-1-SNAP Jurkat cell characterization. **a** Flow cytometry histograms showing PD-1 surface expression levels (top) and quantification (bottom) of indicated Jurkat cells. Based on BD Quantibrite kit, given that PE: antibody is 1:1, number of PD-1 receptors was estimated by dividing the number of antibodies bound by the surface area (11.5 µm diameter, assuming a non-convulated membrane[1]). **b** Western blots of PD-1 and SNAP-tag expressions in cell membrane lysates and corresponding Ponceau S stained blots of total protein content. **c** IL-2 secretion of PD-1-SNAP cells stimulated with antibodies and PD-L1 proteins. Data are presented as mean (cross) ± standard error of the mean from three independent experiments (N = 3), with each run in triplicates. *$P < 0.05$, **$P < 0.01$; one-way ANOVA, Tukey HSD.

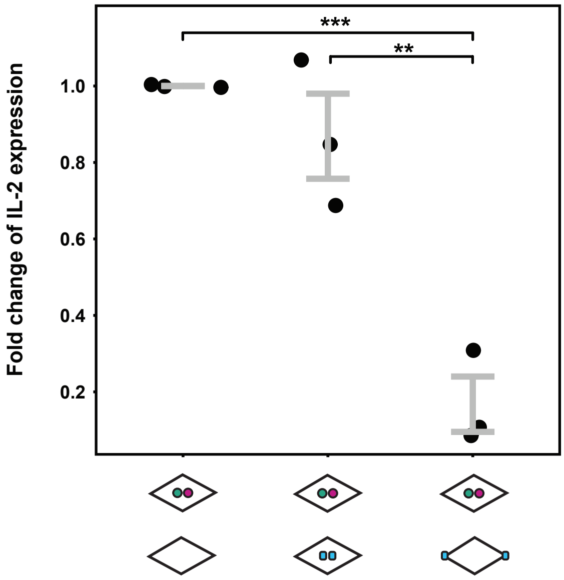

Figure S20. IL-2 gene expression in PD-1-SNAP cells stimulated with antibody and PD-L1 flat sheets by qRT-PCR. Conditions are 0.5 nM FS-α-CD3-CD28 and 5 nM FS-empty (positive control), 0.5 nM FS-α-CD3-CD28 and 5 nM FS-PD-L1-13 and 0.5 nM FS-α-CD3-CD28 and 5 nM FS-PD-L1-200. Fold changes of gene expression are calculated relative to FS-α-CD3-CD28. Data is presented from three independent experiments (N = 3) as indicated by the black dots, with each run in duplicates. ** $P< 0.01$, *** $P< 0.001$, one-way ANOVA, Tukey HSD.

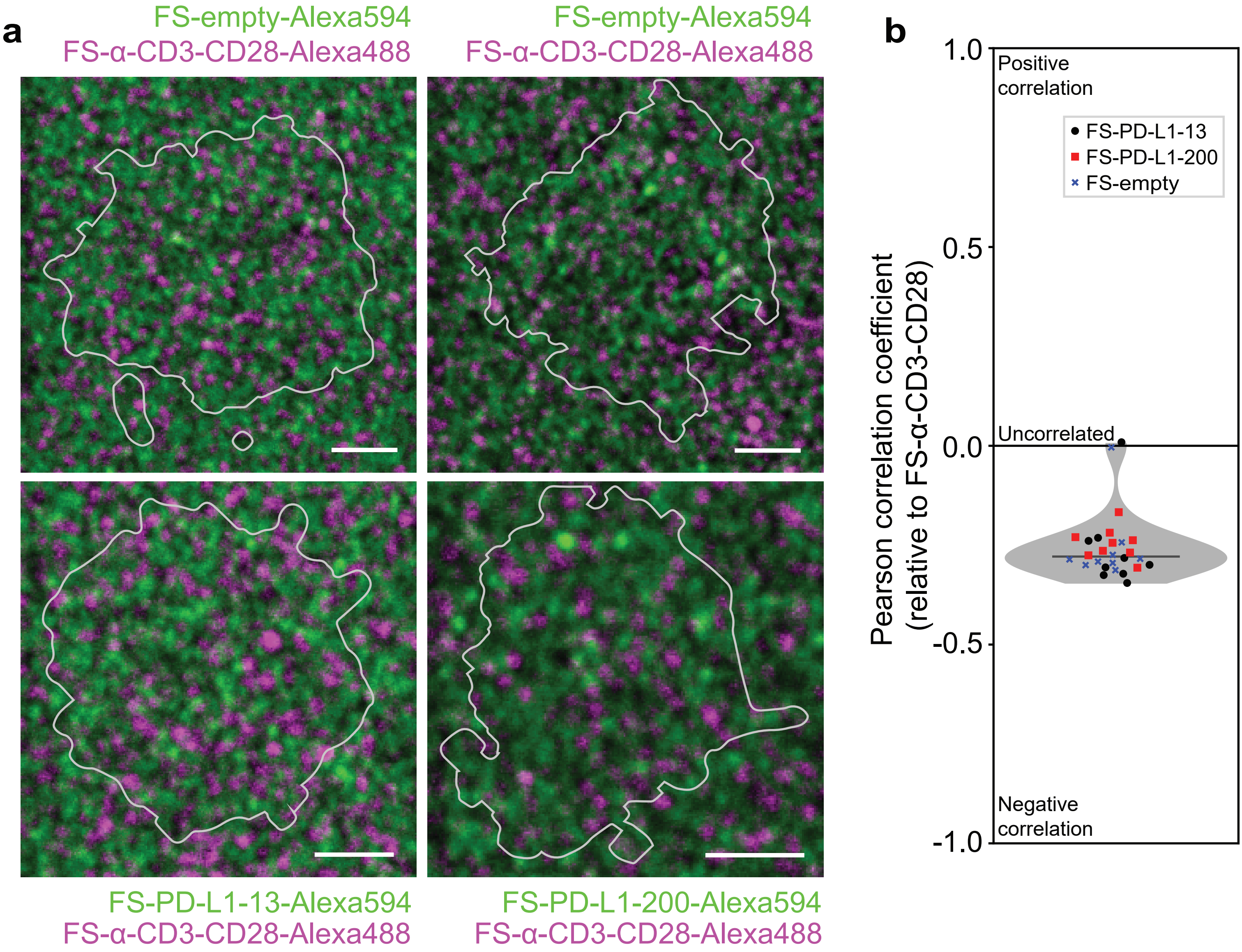

Figure S21. Confocal imaging and colocalization analysis of FS-α-CD3-CD28, FS-empty, FS-PD-L1-13 and FS-PD-L1-200. **a** Representative confocal images showing fluorescently-labeled origami sheets. FS-α-CD3-CD28 (Alexa Fluor 488, magenta), FS-empty, FS-PD-L1-13 and FS-PD-L1-200 (Alexa Fluor 594, green) were simultaneously imaged together with SNAP-647-SiR-labeled PD-1 on the cells (gray outline). Scale bars = 2 µm. **b** Correlation analysis of the two-colour origami sheets. The graph shows the overall measured population of Pearson correlation coefficients for the images (gray shaded area), the overall median (gray bar) and the individual image correlation coefficients of the three different types of samples (FS-PD-L1-13: black dots, FS-PD-L1-200: red squares, FS-empty: blue crosses). N = 27 images taken at cell positions have been analysed.

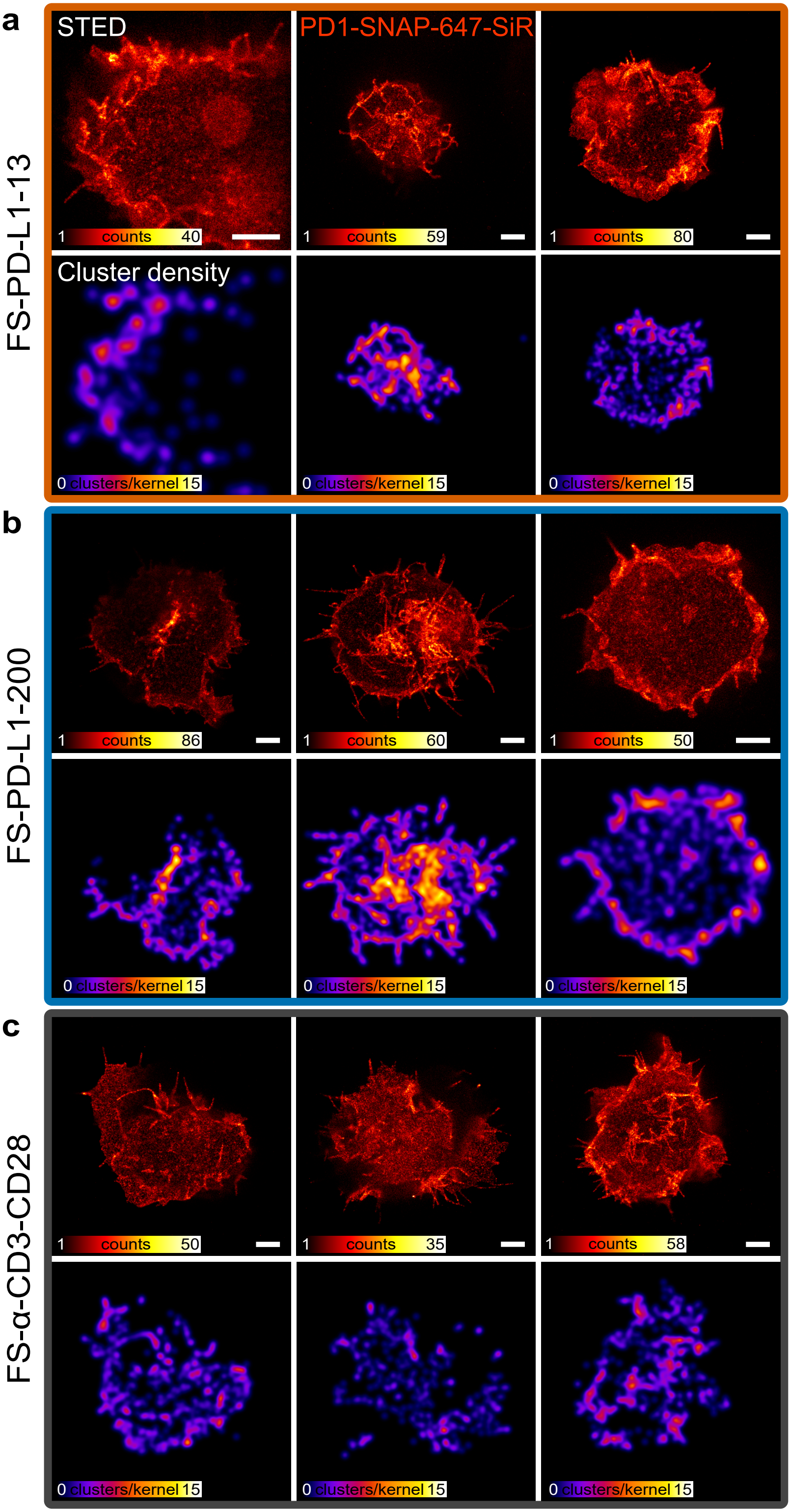
a
STED
PD1-SNAP-647-SiR
1 counts 40
1 counts 59
1 counts 80
Cluster density
0 clusters/kernel 15
0 clusters/kernel 15
0 clusters/kernel 15
FS-PD-L1-13
b
1 counts 86
1 counts 60
1 counts 50
0 clusters/kernel 15
0 clusters/kernel 15
0 clusters/kernel 15
FS-PD-L1-200
c
1 counts 50
1 counts 35
1 counts 58
0 clusters/kernel 15
0 clusters/kernel 15
0 clusters/kernel 15
FS-α-CD3-CD28

Figure S22. Representative raw STED images and cluster density maps of cells stimulated with FS-PD-L1-13, FS-PD-L1-200 or FS-α-CD3-CD28. **a-c** Raw STED images (top row) in which PD-1 was labeled with SNAP-647-SiR and corresponding cluster density maps (bottom row),of (**a**) FS-PD-L1-13 (orange box), (**b**) FS-PD-L1-200 (blue box), and (**c**) FS-α-CD3-CD28 (gray box). Scale bars, 2 μm.

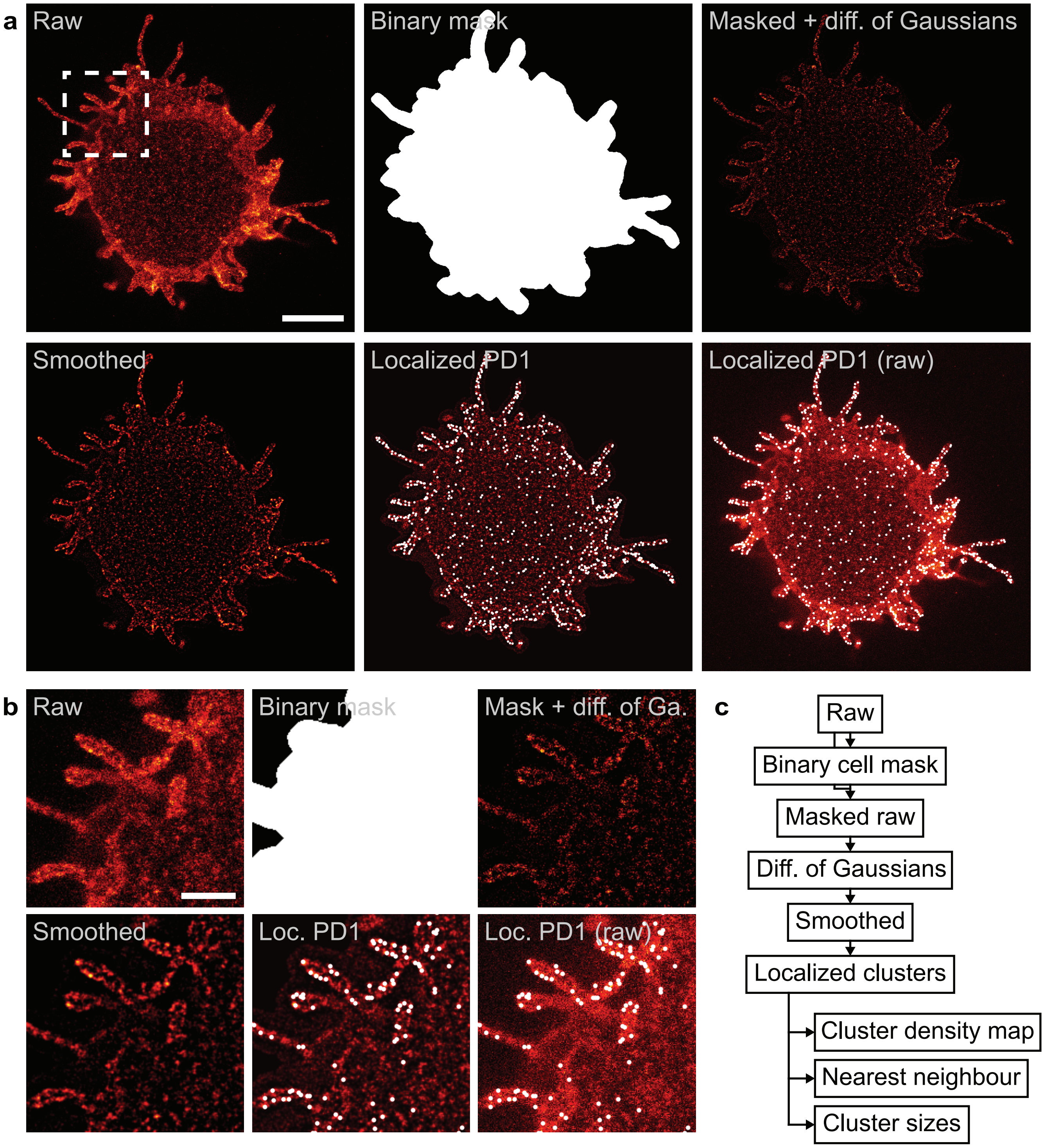

Figure S23. STED image pre-processing and cluster localization pipeline. **a** A pre-processing example of a whole cell in the following steps: raw STED image (top left), binary mask (top center), masked difference of Gaussians image (top right), smoothed difference of Gaussians image (bottom left), localized PD-1 receptors (white circles) overlaid on smoothed difference of Gaussians image (bottom center), and localized PD-1 receptors (white circles) overlaid on raw STED image (bottom right). Scale bar, 3 μm. **b** Zoom-in from the region of interest marked in **a**, with the same pre-processing steps as in **a**. Scale bar, 1 μm. **c** Pipeline diagram of pre-processing, receptor localization, and results (cluster density map, nearest neighbour analysis, and cluster size analysis). Image example is a cell stimulated with FS-PD-L1-200.

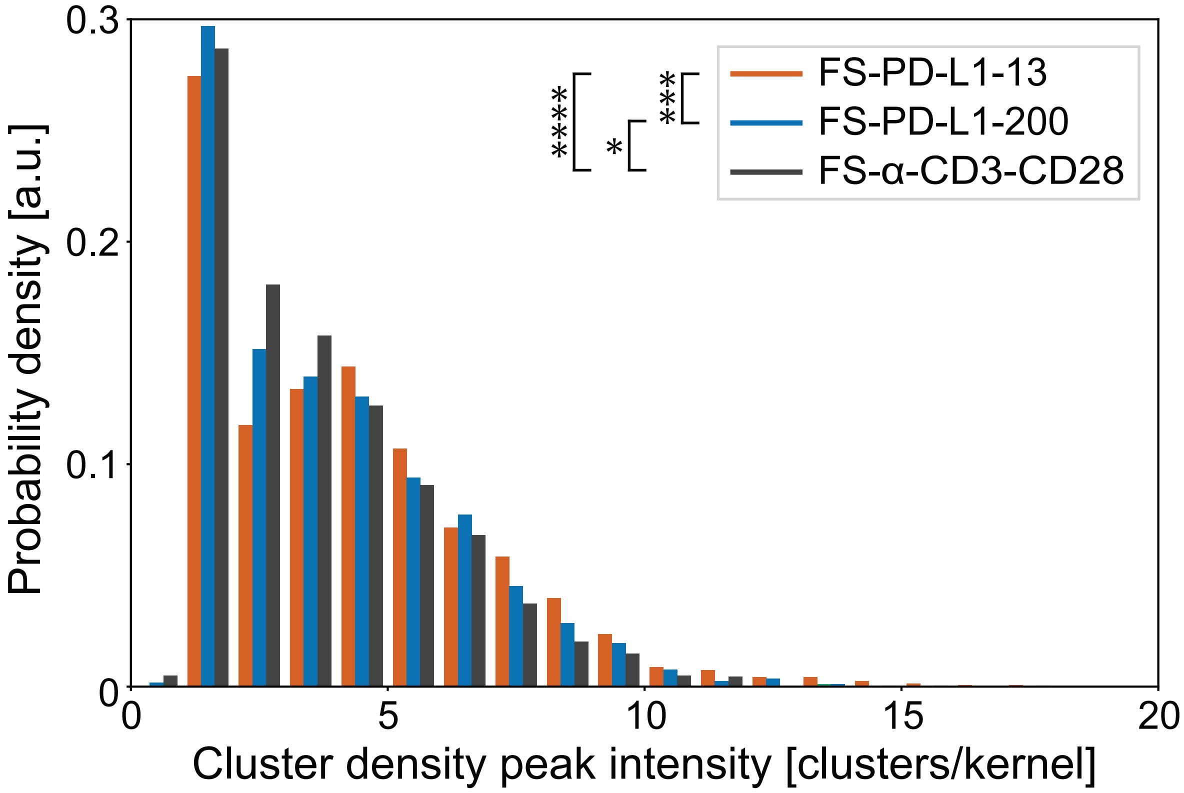

Figure S24. Cluster density peak intensity pooled histogram**.** Histogram of cluster density peak intensity distributions from pooled data for the three different conditions. Each distribution contains the peak intensities from N=24 cells of respective condition. Reported significances P < 0.05 (*), P < 0.01 (**), P < 0.001 (***) and P < 0.0001 (****) are from one-sided Kolmogorov-Smirnov tests.

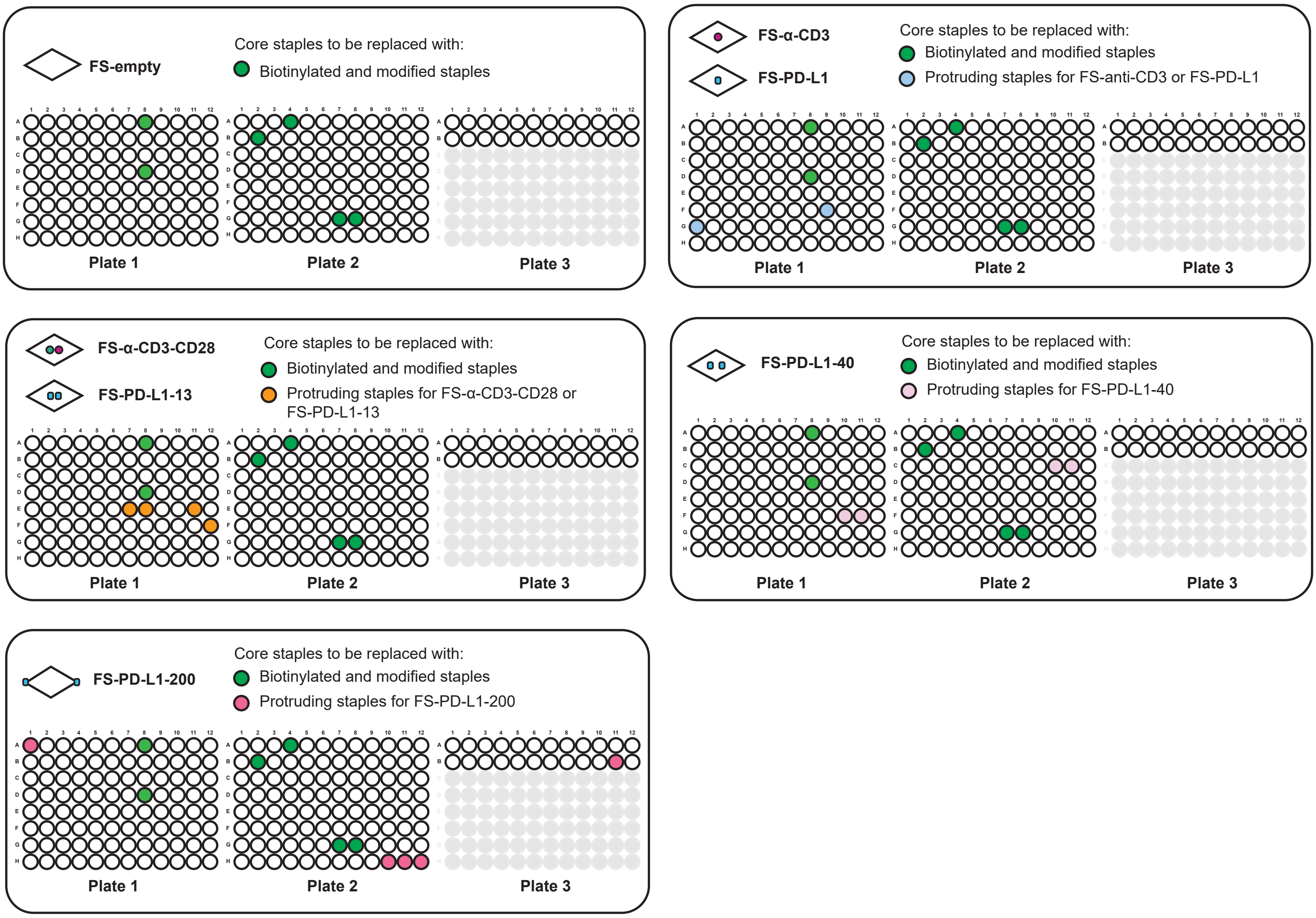

Figure S25. Pipetting schemes of staple mixes for producing the different flat sheet designs. The biotinylated flat sheet design requires a total of 216 staples which includes a pool of core staples (white wells) of which six are replaced by biotinylated and modified staples (green wells). To generate flat sheets for protein hybridization, some of the core staples are replaced with protruding staples (orange, blue, light pink and pink wells).
